# Adsorption of Pharmaceutical Contaminants from a Real Hospital Effluent Using Açaí Biomass-Derived Biochar Modified via Magnetron Sputtering

**DOI:** 10.1021/acsomega.6c05238

**Published:** 2026-08-18

**Authors:** Ronaldo Antunes Funari Junior, Sabrina Frantz Lütke, Luis Felipe Oliveira Silva, Marcos Leandro Silva Oliveira, Alex Castro, Guilherme Luiz Dotto

**Affiliations:** † 28118 Universidade Federal de Santa Maria, Santa Maria, State of Rio Grande do Sul 97105-900, Brazil; ‡ 27951 Corporación Universitaria de la Costa, Barranquilla 080002, Colombia; § 245076 Universidade Federal do Oeste do Pará, Santarem, Pará 68035-110, Brazil

## Abstract

A novel aluminum-coated biochar derived from açaí waste was successfully engineered via physical vapor deposition (magnetron sputtering) to remove the emerging pharmaceutical contaminants clorazepate (Clz) and diclofenac (Dic) from synthetic and real aqueous systems. Aluminum deposition at 200 W significantly altered the biochar’s surface morphology and chemistry, introducing Al-related active sites that enhanced interactions with anionic drug species. Kinetic studies revealed that the PSO model best describes the data (*R* ^2^ = 0.995 for Clz and 0.993 for Dic), with rapid adsorption equilibrium reached within 120 min for Clz and 80 min for Dic. Equilibrium data were best fitted by the Sips model, indicating heterogeneous adsorption. Increasing temperature from 298 to 328 K enhanced the maximum adsorption capacity (*Q*_ms_) from 260 to 437.8 mg g^–1^ for Clz and from 277 to 442.8 mg g^–1^ for Dic. Thermodynamic parameters confirmed spontaneous adsorption and a weakly exothermic process. The application in a real hospital wastewater demonstrated 95% removal of Dic and >88% removal of other pharmaceuticals, reducing total drug concentration from approximately 50–5 mg L^–1^. Regeneration tests showed stable performance in the sixth cycle (250–270 mg g^–1^), with a gradual loss of efficiency in subsequent cycles due to partial aluminum leaching. These results highlight AlAB_200W as a promising, high-capacity, and scalable adsorbent for pharmaceutical remediation in complex aqueous matrices.

## Graphical abstract

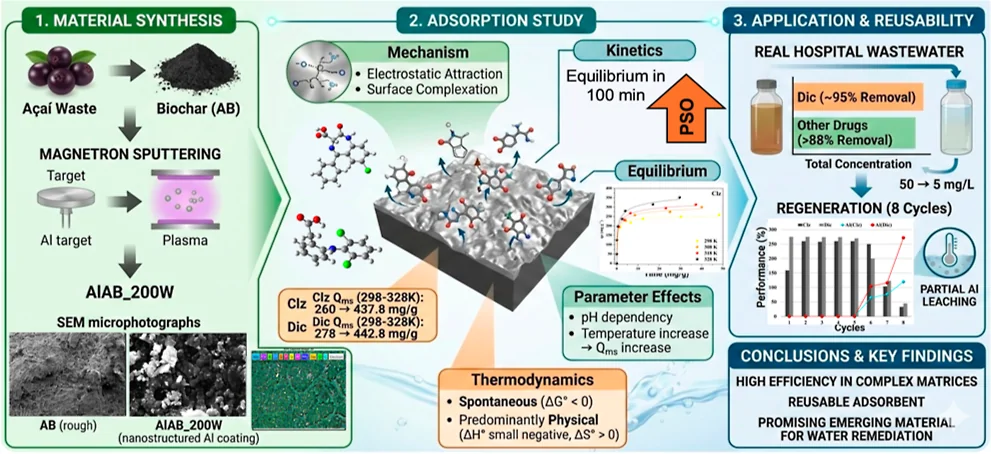

## 1 Introduction

Effective solutions for removing drug contaminants have gained significant attention over the last few decades due to their impact on human and environmental health.^1^ The rapid advancement of modern medicine has led to a significant increase in the production and consumption of pharmaceuticals, consequently intensifying the generation of pharmaceutical waste.^2^ Although the expansion of healthcare services has significantly improved human well-being in recent decades, it has also created a major problem: large-scale wastewater generation.^3,4^ In addition to conventional pollutants, hospital effluents are heavily contaminated with a wide range of pharmaceutically active compounds (PhACs) and their metabolic byproducts. These compounds often include various medications, such as antibiotics, analgesics, anticonvulsants, and substances that interfere with endocrine function.^5^ The release of these wastes into the environment poses a serious threat to aquatic life and fosters the emergence of drug-resistant pathogens, potentially facilitating the spread of more dangerous and virulent diseases.^6,7^

Among these PhACs, clorazepate (Clz) is one of the most widely used benzodiazepines, while diclofenac (Dic) is a predominant nonsteroidal anti-inflammatory drug (NSAID) frequently detected in the environment.^8–12^ Clz is frequently used as an anxiolytic, hypnotic, sedative, and anticonvulsant agent, indicating its widespread use to manage pain and inflammation.^13,14^ These drugs have been found in water bodies globally, and their persistence in aquatic ecosystems raises concerns about potential negative impacts on human health and aquatic organisms.^1,15^ Among benzodiazepine drugs, Clz has a concentration of 6.227 ng L^–1^ and exhibited a significant increase in total offspring when exposed to drug levels as much as 273 μg L^–1^.^16–18^ Additionally, Dic disposal ranges from 0.1 μg L^–1^ to 2.1 μg L^–1^. In contrast, a significant concentration of 7.1 μg L^–1^ of Dic has been found in water effluent, exceeding the 0.1 μg L^–1^ environmental quality standard for Dic in water.^19,20^

Effective treatment of hospital wastewater remains a major challenge due to its role in disseminating several pharmaceuticals into the environment, posing potential risks to public health.^21^ Consequently, countries such as China and Japan have implemented on-site pretreatment of hospital wastewater to reduce the release of pathogenic microorganisms into municipal sewer systems.^21,22^ In this way, researchers have been actively exploring several environmentally friendly methods for removing Clz, Dic, and other pharmaceutical pollutants from water sources, such as photocatalysis, advanced oxidation processes, and adsorption.^23–26^

Adsorption is a low-cost and powerful method to remove pollutants from various water matrices, and a wide range of cost-effective adsorbent materials has been explored, including graphene-based oxides, engineered nanoparticles, zeolites, and, notably, biochar.^27–29^ In Brazil, the large-scale processing of açaí fruit generates substantial quantities of residual fibers and seeds, which are often discarded, creating an environmental burden.^30,31^ These lignocellulosic residues can be valorized through pyrolysis under oxygen-limited conditions, yielding a carbon-rich solid with well-developed porosity and considerable surface area.^32^ Owing to its low production cost, scalability, and adaptable surface chemistry, biochar has gained increasing attention as a competitive alternative to conventional adsorbents, particularly when surface modifications are introduced to tailor its physicochemical characteristics and enhance adsorption performance.^33,34^

Recent studies have demonstrated the potential of modified biochars for the adsorption of antibiotics from hospital wastewater. For instance, NaOH-activated forest biomass biochar achieved complete Meropenem removal at low concentrations while maintaining high performance after multiple regeneration cycles.^21^ Likewise, a magnetic biochar functionalized with MnFe_2_O_4_ nanoparticles and a Cu@Zn-BDC metal–organic framework exhibited enhanced tetracycline adsorption capacity and excellent reusability, highlighting the benefits of surface engineering for improving adsorption performance.^35^ Furthermore, a recent review identified metal-modified biochars as promising adsorbents for hospital wastewater treatment due to their enhanced surface functionality and adsorption efficiency, and emphasized the need to develop highly reusable materials capable of removing a broader range of pharmaceutical contaminants under realistic wastewater conditions.^36^

Among the available modification strategies, magnetron sputtering has recently emerged as a promising approach for biochar functionalization.^19,28–30^ This physical vapor deposition (PVD) technique relies on plasma-assisted ejection of atoms from a solid target, which are subsequently deposited as a uniform coating on the adsorbent surface, allowing controlled surface modification and uniform thin-film formation without extra chemical reagents.^28,37–39^ Besides that, among available deposition techniques, magnetron sputtering is a preferable technique for many industrial processes due to its scalability, versatility, and high reproducibility.^40,41^ Despite the high operational costs of the magnetron sputtering technique, it does not require chemicals or solvents, making it less waste-generating and ultimately mitigating the costs incurred in treating secondary waste.^41–43^

Despite its potential to significantly improve adsorption efficiency, the application of magnetron sputtering in this context remains relatively underexplored, with only a limited number of studies addressing its use for removing emerging contaminants, such as textile, mining, and pharmaceutical residues.^44–47^ In our previous study,^35^ different adsorbents were developed via magnetron sputtering under varying time and power conditions. The conditions for magnetron sputtering were optimized to identify a better adsorbent for Dic and Clz. It was concluded that Al-covered biochar produced at 200 W for 15 min was the best. However, the adsorption kinetics and isotherms were evaluated only at a single condition, and the thermodynamics were not evaluated. Besides, the adsorption was carried out only in synthetic solutions. Therefore, the best adsorbent previously developed was applied here in an integrated kinetic, isothermal, thermodynamic, and hospital real-sample study.

This study explores the use of magnetron sputtering to deposit an aluminum thin film onto biochar derived from açaí waste to enhance the adsorption of the drugs Clz and Dic. The optimized sputtering power and deposition time have been adjusted, resulting in a novel Al-coated biochar material. A comprehensive characterization and adsorption studies were conducted, including adsorption from real hospital wastewater, to evaluate the mechanisms and capacity of the novel Al-coated material. The adsorption of the Al-modified material was assessed to verify its suitability as a robust, reusable adsorbent for eliminating Clz and Dic from aqueous media.

## 2 Materials and Methods

### 2.1 Synthesis of Aluminum-Coated Biochar

The raw biochar was previously synthesized from açaí seeds. First, the seeds were sun-dried and ground to obtain a particle size less than 1 mm. Afterward, the seeds were placed in a tubular quartz reactor, which was inserted into a furnace for pyrolysis. Finally, pyrolysis was performed at 700 °C under a flow of 25 mL min^–1^ of N_2_ for 1 h. These conditions were determined from preliminary tests.

An aluminum target with 99.99% purity was used. Aluminum deposition was performed by PVD using magnetron sputtering (Figure 1), at a sputtering power of 200 W for 15 min, and a working pressure of 3 × 10^–3^ mbar. This procedure resulted in the formation of a thin aluminum film on the biochar surface, which was named AlAB_200W.^48,49^

**Figure 1:**
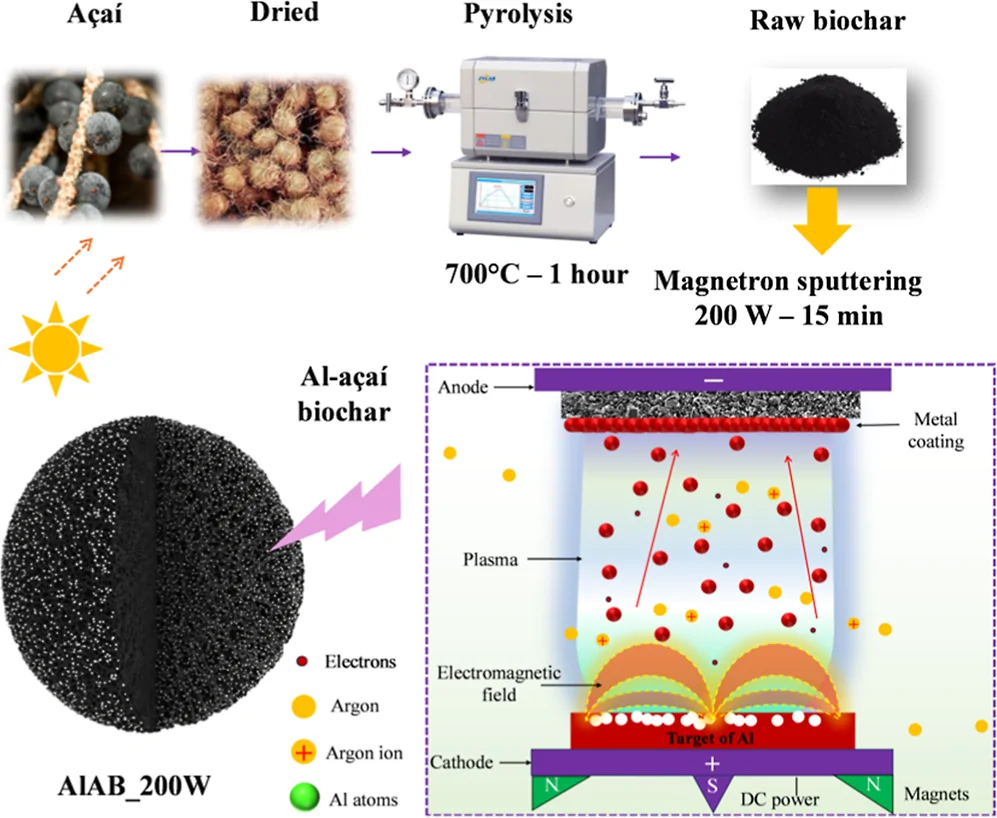
Schematic synthesis of AlAB_200W biochar from açaí.

### 2.2 Characterization of the AlAB_200W Biochar

A suite of analytical techniques was used to characterize the novel material. Scanning electron microscopy and energy dispersive X-ray spectroscopy (SEM-EDS) were used to examine the morphology and elemental distribution. Further elemental analysis was conducted via X-ray fluorescence (XRF). The material’s crystalline structure and functional groups were determined by X-ray diffraction (XRD) and Fourier transform infrared (FTIR) spectroscopy, respectively. The zero-point charge was determined by the 11-point method. X-ray photoelectron spectroscopy (XPS) measurements were performed using a SPECS system equipped with a PHOIBOS 150 1D-DLD hemispherical electron analyzer. Lastly, the textural characteristics, such as specific surface area, pore volume, and pore size, were determined using N_2_ porosimetry (BET and BJH approaches). All instrumentation was provided by the Research Group on Adsorptive and Catalytic Process Engineering and its collaborating partners.

### 2.3 Adsorption Kinetics, Equilibrium, and Thermodynamic Experiments

Stock solutions of Clz and Dic were prepared at 1000 mg L^–1^ in distilled water. The kinetic study for Clz and Dic was performed at a *C*_0_ of 25 mg L^–1^, a dosage of 0.2 g L^–1^, and pH = 3 for 4 h. The kinetic models applied were pseudo-first-order (PFO), pseudo-second-order (PSO), and Elovich models (eqs 1–3).^50,51^

1 $$q_{t}=q_{e}\left(1-e^{-k_{1}t}\right)$$

2 $$q_{t}=\frac{{qe}^{2}k_{2}t}{1+k_{2}qet}$$

3 $$q_{t}=\frac{1}{\beta }\ln\left(\alpha \beta t\right)$$

where *q*_e_ is the equilibrium adsorption capacity, *k*_1_ and *k*_2_ are the PFO and PSO constants, α is the adsorption rate, and β is the desorption constant.

Equilibrium adsorption experiments for Clz and Dic onto AlAB_200W biochar were performed in batch mode (50 mL volume, 0.2 g L^–1^ dosage) at different temperatures (298–328 K) and pH 3.0. Initial solute concentrations were systematically varied from 0 to 100 mg L^–1^ to generate isotherm data. The equilibrium adsorption behavior was evaluated by the monolayer Langmuir model, the multilayer Freundlich model, and the monolayer and multilayer Sips models (eqs 4–6).^52,53^

Thermodynamic parameters enthalpy change (Δ*H*°), entropy change (Δ*S*°), and Gibbs free energy change (Δ*G*°) were determined in accordance with eqs 7–9.^54^

4 $$q_{e}=\frac{q_{m}K_{L}C_{e}}{1+K_{L}C_{e}}$$

5 $$q_{e}=K_{F}C_{e}^{1/n}$$

6 $$q_{e}=\frac{q_{ms}{\left(K_{s}C_{e}\right)}^{m}}{1+{\left(K_{s}C_{e}\right)}^{m}}$$

7 $$K_{e}=\frac{K\gamma^{M}}{\gamma }$$

8 $${\ln K}_{e}=\frac{-\Delta H^{{}^{\circ}}}{R}\frac{1}{T}+\frac{\Delta S^{{}^{\circ}}}{R}$$

9 $$\Delta G^{{}^{\circ}}=-RT\ln\left(K_{e}\right)$$

where *q*_m_ and *q*_ms_ are the maximum adsorption capacity of the Langmuir and Sips isotherm models, respectively, *K*_L_, *K*_F_, and *K*_s_, are isotherm-related constants of the Langmuir, Freundlich, and Sips models, respectively, 1/*n* is the heterogeneity factor of the Freundlich model, *q*_m_ is the exponent of the Sips model, *K*_e_ represents the thermodynamic constant, γ and γ^*M*^ (1 mol L^–1^) denote the activity coefficients, *T* is the temperature, and *R* is the universal gas constant. Model fitting and calculations were carried out using OriginPro 2025 (64-bit), and the statistical parameters *R* ^2^, *R*_adj_^2^, and RMSE were used to determine the best-fitting models.

### 2.4 Real Hospital Sample Wastewater Analysis and Experiments

Real hospital wastewater was collected from the University Hospital of Santa Maria (HUSM), located in Santa Maria, Brazil. Sampling was done in triplicate at a single point in May 2025. Adsorption experiments were conducted directly using the untreated effluent, without dilution or prior processing, under the following conditions: 298 K, adsorbent dosage of 0.2 g L^–1^, original pH of the hospital wastewater, and a contact time of 2 h, using AlAB_200W. Removal percentages were calculated for each pharmaceutical compound individually according to eq 10.

10 $$R=\frac{\left(C_{0}-C_{e}\right)}{C_{0}}100$$

where *R* is the removal percentage and *C*_0_ and *C*_e_ are the initial and equilibrium concentrations of each pharmaceutical compound (mg L^–1^).

Physicochemical characterization included the determination of total organic carbon (TOC), nonpurgeable organic carbon (NPOC), and total nitrogen using a Shimadzu TOC-LCPH/CPN analyzer. Chemical oxygen demand (COD) was measured in accordance with ISO 6060:1989. The quantification of pharmaceutical compounds before and after adsorption was carried out in the same manner as Vieira et al. (2021).^55^ Accordingly, due to the high concentration of the analytes in the analyzed wastewater, the method’s sensitivity exceeded the requirements. Uncertainties were smaller than 30% at a confidence interval of 95%, and the relative recovery was between 83 and 115% in the matrix.

### 2.5 Desorption and Reusability

The desorption and reusability of AlAB_200W biochar were assessed over eight successive adsorption–desorption cycles. Experiments were conducted using 1.0 g of adsorbent in 50 mL of 0.5 mol L^–1^ NaOH, under agitation at 200 rpm for 1 h at 298 K. Following each desorption step, the solid phase was separated by centrifugation, and the resulting supernatant was collected for the determination of Clz and Dic.^32^ Residual aluminum in the solution was determined by atomic absorption spectroscopy (Agilent Technologies, model 240 FS AA), using an acetylene flame and compressed air as the oxidant.

## 3 Results and Discussion

### 3.1 Features of AlAB_200W Biochar

This study obtained a biochar coated with an aluminum thin film (AlAB_200W) via magnetron sputtering to adsorb the pharmaceuticals Clz and Dic. Figure 1 shows a schematic illustration of the AlAB_200W synthesis.

The FTIR spectra of the raw and aluminum-modified biochars (AlAB_200W) are shown in Figure 2. Distinct stretching vibrations appear in the modified biochar, indicating that aluminum coating enhances surface functionalization and, consequently, the potential for interaction with Clz and Dic molecules. The broad absorption band centered around 3445 cm^–1^ corresponds to O–H stretching vibrations, whose increased intensity upon aluminum deposition suggests the formation of hydroxyl functionalities on the modified biochar surface.^37^ The weak band near 2950 cm^–1^ is attributed to CH_2_ stretching vibrations, which are commonly observed in carbonaceous materials.^56,57^ The absorption bands around 1640 cm^–1^ show aromatic C=O stretching or C=C bonds generated during pyrolysis.^58^ These features originate from lignin structures (C=O) and hemicellulose.^30,59^ Vibrations detected within the 1000–500 cm^–1^ range correspond to specific molecular modes. Normally, metallic species cause spectral distortion near 600 cm^–1^ (Figure 2); however, the low intensity and proximity to baseline noise imply that aluminum is primarily deposited on the biochar surface rather than forming strong chemical bonds.^60,61^

**Figure 2:**
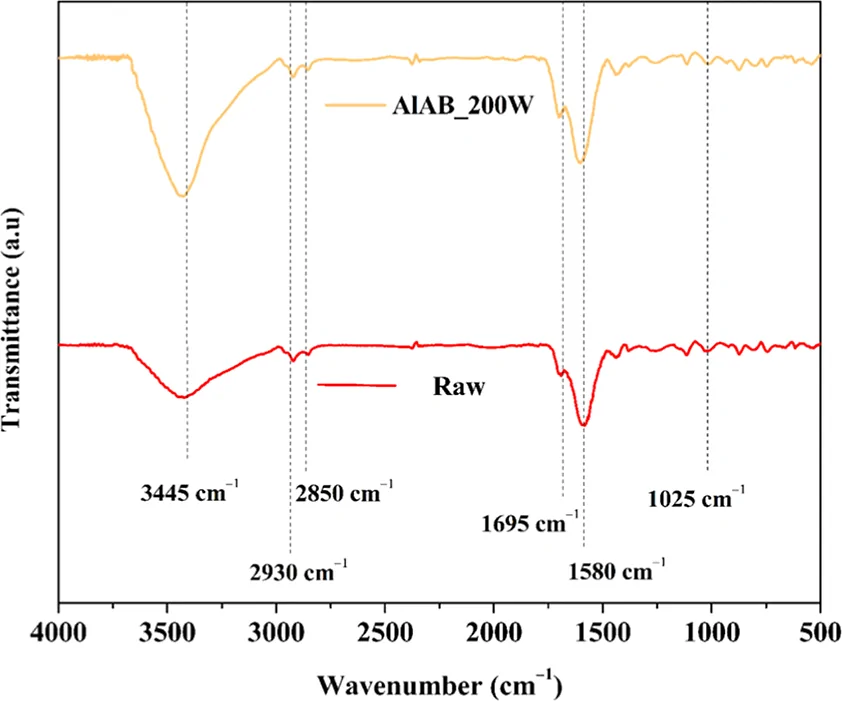
FTIR of raw and AlAB_200W biochar.

The surface morphology of the coated biochar at different magnifications is shown in Figure 3a–d. The adsorbent exhibited changes in its surface after aluminum deposition by magnetron sputtering. The raw biochar displayed distinct surface grooves characteristic of carbonaceous materials (Figure S1). In contrast, the aluminum-coated sample showed surface deformation with 200 W of deposition power (Figure 3). The AlAB_200W biochar surface exhibited localized irregularities, indicating initial aluminum accumulation and a higher density of aluminum particles, suggesting enhanced deposition. At 200 W, a continuous aluminum layer formed with numerous dispersed particles (bright dots) covering the surface, confirming successful thin-film formation.^61,62^ These morphological features are consistent with those reported for other coal-based carbon materials.^31,63,64^

**Figure 3:**
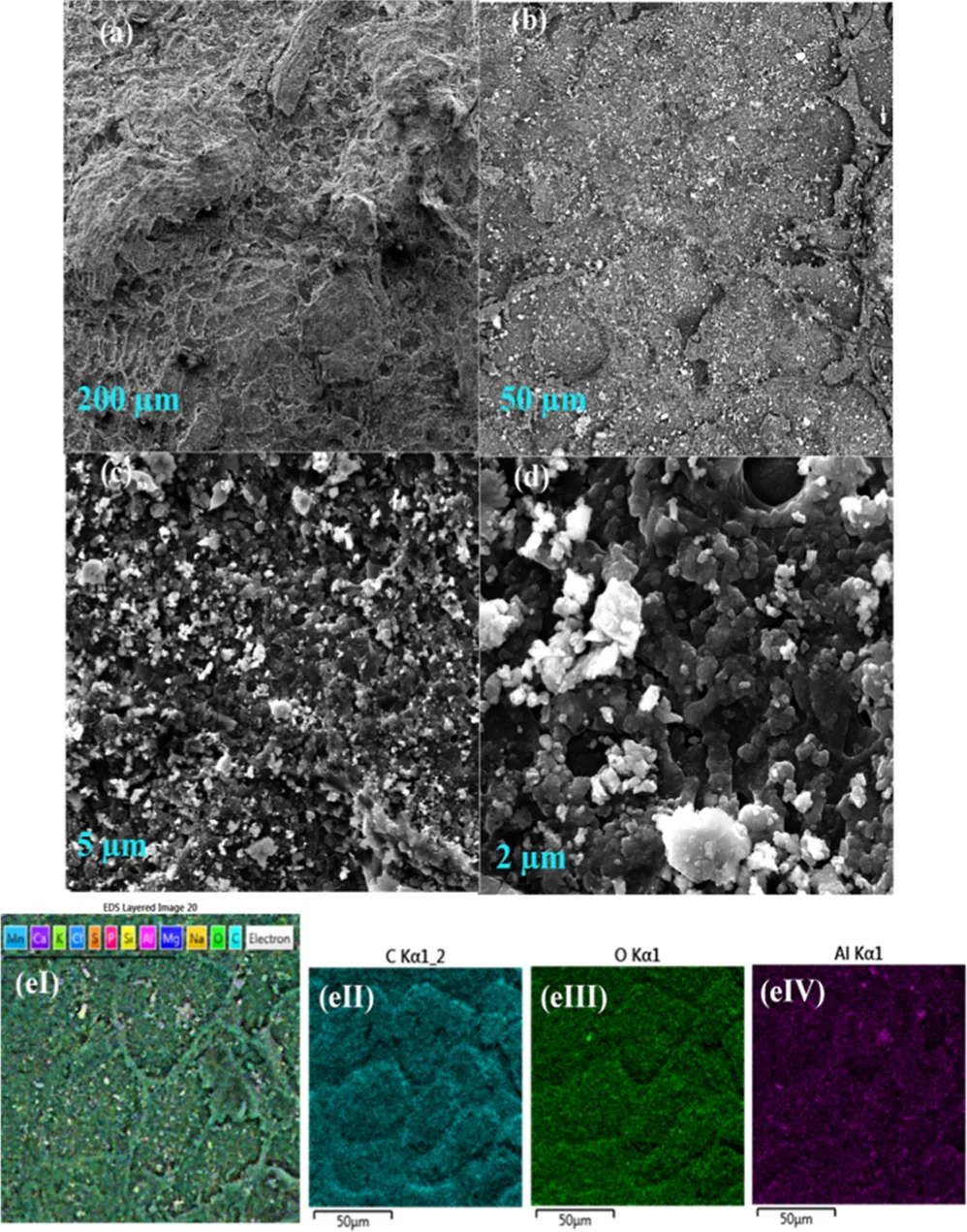
(a–d) SEM images and (eI–IV) EDS mappings of AlAB_200W biochar.

Elemental mapping corroborates these findings (Figure 3eI–IV). The EDS mappings obtained for AlAB_200W revealed a uniform, intense pink color corresponding to aluminum, confirming the effective, homogeneous deposition of an aluminum film across the biochar surface. In contrast, the unmodified biochar predominantly contained carbon and oxygen with trace amounts of silica, calcium, and other minor elements.

The XRD patterns of raw and AlAB_200W biochar are presented in Figure 4. The diffraction peaks at approximately 2θ = 25° indicate the dominance of amorphous regions across all biochar samples. This observation aligns with the typical structural characteristics of carbonaceous materials produced by biomass pyrolysis, which generally exhibit low crystallinity.^65^ The predominance of amorphous phases can be attributed to the intricate and heterogeneous carbon framework of the biochar matrix. Moreover, the slight diffraction signal detected near 2θ = 50° suggests a possible structural modification associated with the deposition of aluminum thin films.^44^

**Figure 4:**
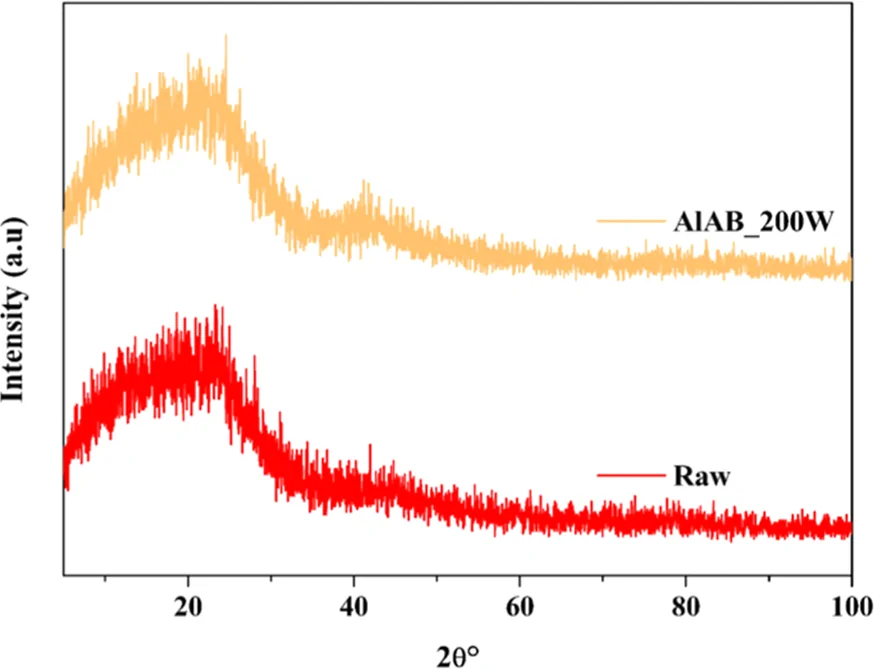
XRD analysis of raw and AlAB_200W biochar.

XRF analysis was also carried out to obtain the elemental compositions. The results for the raw biochar were primarily carbon (76.31%) and oxygen (23%), with trace amounts (<0.07%) of Mg, Si, P, S, K, and Ca. In contrast, the AlAB_200W biochar exhibits a slight decrease in C and O percentages (70.9 and 21.9%) and an increase mainly in the Si percentage (0.90%). Moreover, the Al content was 3.42%, confirming successful deposition of Al on the biochar surface at 200 W. This result indicates that elevated deposition power increases plasma energy, thereby promoting higher atom and ion densities, which, in turn, facilitate metal–carbon interactions and increase aluminum incorporation into the biochar matrix.^66,67^

The XPS spectra of AlAB_200W biochar were obtained to investigate the surface chemical composition and oxidation states (Figure S2). Consistent with the XRF results, the high-resolution Al 2p spectrum confirmed the successful deposition of aluminum on the biochar surface (Figure S2). A single peak centered at 75.85 eV was observed, corresponding to oxidized Al^3+^ species typically associated with Al_2_O_3_ or hydrated alumina phases.^66^ This binding energy is considerably higher than that of metallic aluminum (72.7–73.0 eV).^49^ Although aluminum oxyhydroxides (AlOOH) may also be present, their contribution cannot be clearly distinguished because of the low intensity of the Al 2p signal (Figure S2).

The BET/BJH results (Table 1) showed that aluminum deposition at 200 W significantly reduced both the specific surface area and pore volume of the biochar. The specific surface area decreased by approximately 42.4 m^2^ g^–1^ (352.7 to 310.3 m^2^ g^–1^) after Al deposition (AlAB_200W), accompanied by a reduction in pore volume from 0.245 to 0.201 cm^3^ g^–1^. This behavior is likely associated with partial obstruction of pores caused by the aluminum coating.^68^ Despite these changes, the average pore diameter remained approximately constant (Table 1), indicating that the mesoporous framework was largely preserved after modification. In addition, the presence of aluminum species may introduce hydroxylated surface sites, which can enhance the material’s surface reactivity and potentially increase its affinity toward Clz and Dic molecules.^30,69^

**Table 1:** BET Results of Raw and AlAB_200W Biochars

adsorbent	*S*_BET_ (m^2^ g^–1^)^a^	pore volume (cm^3^ g^–1^)^a^	pore diameter (nm)^a^
raw	352.7 ± 4.5	0.245 ± 0.012	10.0 ± 1.2
AlAB_200W	310.3 ± 2.8	0.201 ± 0.010	10.5 ± 1.0

a Mean ± standard error for *n* = 3.

Based on the thermogravimetric analysis (TGA) and derivative thermogravimetric (DTG) curves shown in Figure 5, the thermal behavior of the raw biochar and the Al-modified biochar reveals a marked improvement in thermal stability after aluminum sputtering treatment. The raw biochar (black curve) exhibits two main degradation steps, with a significant mass loss occurring between approximately 200 and 600 °C. This decomposition is mainly attributed to the volatilization of surface functional groups and the breakdown of organic carbon structures, resulting in a final residual mass of around 78% at 1000 °C.

**Figure 5:**
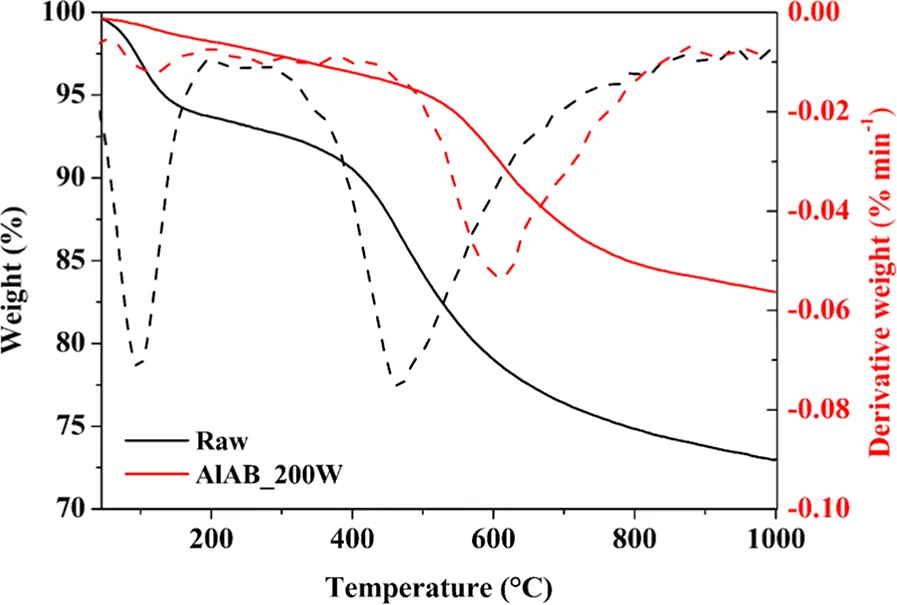
TGA and DTG graph of raw (black lines) and AlAB_200W (red lines).

In contrast, the AlAB_200W sample (red curve) shows a more gradual mass loss throughout the entire heating range, indicating enhanced thermal stability. The total mass loss of the AlAB_200W biochar is notably lower, with a final residual weight of about 86%, suggesting that the aluminum coating helps preserve the carbon matrix. The DTG profile further supports this observation: the maximum mass-loss rate for the raw biochar occurs near 450 °C, whereas for AlAB_200W, the decomposition peak is shifted to higher temperatures, around 600 °C, and the derivative peak intensity decreases significantly. These results demonstrate that the Al layer deposited at 200 W serves as an effective physical barrier, reducing the biochar surface’s exposure to oxidative and thermal degradation. The improved thermal resistance can be attributed to the presence of metal on the surface, which limits heat and mass transfer between the biochar core and the external environment.^61,70^ Consequently, the AlAB_200W biochar exhibits superior structural integrity and thermal robustness compared to the unmodified sample.

### 3.2 Aluminum-Deposition and Contact Time Effect in Clz and Dic Adsorption

Since Clz and Dic removal can be strongly influenced by the adsorbent’s surface properties, making a comparison between the raw and AlAB_200W biochar is essential for determining the optimal material. As shown in Figure 6, the Al-coated biochar exhibited significantly higher adsorption capacities for both pharmaceuticals throughout the entire contact time range (0–90 min). For Clz and Dic, the AlAB_200W biochar achieved adsorption values almost twice as high as those of the Raw biochar at all measured times, demonstrating the pronounced effect of surface modification on adsorption performance.

**Figure 6:**
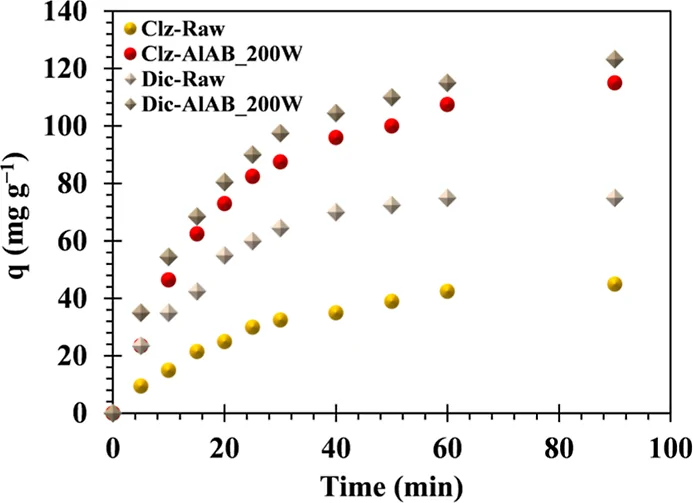
Comparison of raw and AlAB_200W biochar at different times.

This improvement is attributed to the enhanced surface chemistry of the Al coating, which increases the density of reactive oxygen-containing groups and improves surface charge characteristics. These features promote stronger electrostatic interactions and facilitate surface complexation with both pharmaceutical molecules, leading to faster uptake rates and higher equilibrium adsorption capacities. In contrast, the raw biochar displayed a slower kinetic profile and substantially lower adsorption capacity, consistent with its more hydrophobic character and limited availability of functional groups. In addition, for both molecules, the equilibrium was approached within 90 min, as indicated by the plateau in *q* values for AlAB_200W. This rapid stabilization reflects the high accessibility of active sites and the efficient interaction between the coated surface and the adsorbates. Thus, the superior kinetic behavior and adsorption capacity observed for the AlAB_200W material highlight the beneficial effect of Al deposition on biochar reactivity. For this reason, AlAB_200W biochar was selected for further adsorption experiments.

### 3.3 Effect of pH on Clz and Dic Adsorption onto AlAB_200W

The effect of pH on the adsorption of Clz and Dic was evaluated over a wide range (pH 1–8) at an initial concentration of 100 mg L^–1^ using the AlAB_200W biochar. Clz and Dic have p*K*_a_ values of 3.5 and 4.0, respectively, meaning that both contaminants predominantly exist in their protonated forms under acidic conditions, whereas their deprotonated species become dominant at higher pH. These characteristics help to explain the adsorption trends observed in Figure 7a. At strongly acidic conditions (pH = 3), the adsorption capacities are relatively low, likely due to charge similarity between the protonated adsorbates and the positively charged biochar surface, which weakens electrostatic attraction. As pH increases from 2 to 3, the adsorption performance improves markedly, reaching approximately 120 mg g^–1^ for both Clz and Dic. This enhancement is consistent with conditions near the p*K*_a_ values, where partial deprotonation leads to the emergence of lightly negative adsorbate species, which interact more favorably with the slightly positive Al-coated surface.

**Figure 7:**
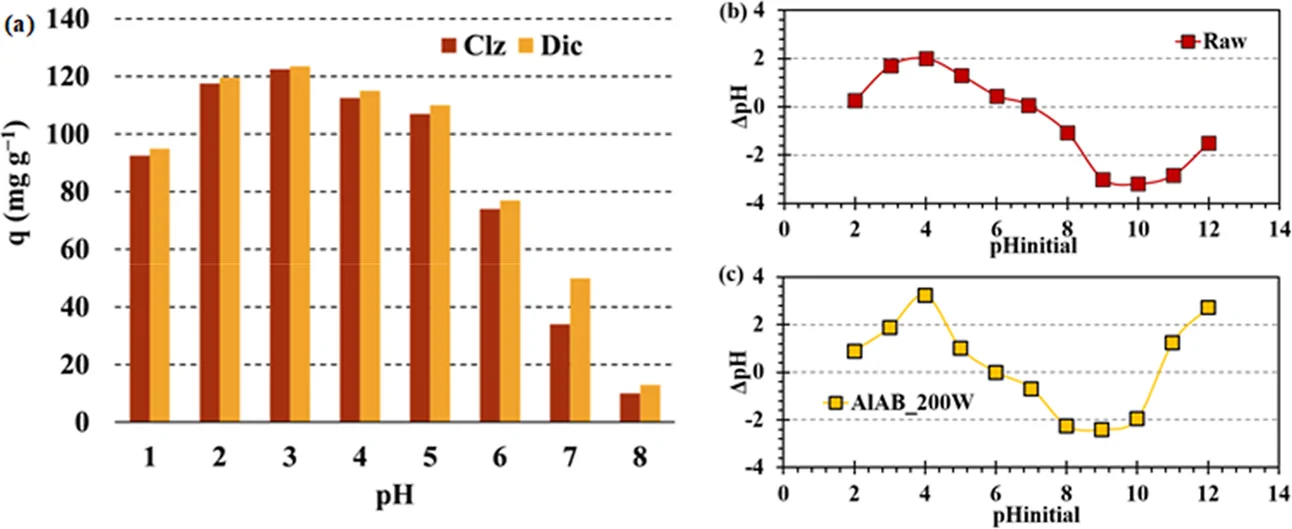
(a) Effect of pH on Clz and Dic adsorption on AlAB_200W biochar, and point of zero charge of (b) raw biochar and (c) AlAB_200W biochar.

Beyond this point, increasing the pH from 4 to 8 results in a continuous decline in adsorption capacity, from about 110 mg g^–1^ to roughly 11 mg g^–1^, reflecting the increasing prevalence of negatively charged adsorbate species. These findings align with the point of zero charge of AlAB_200W (PZC = 6.0) (Figure 7b,c), indicating that at pH > 6 the biochar surface becomes predominantly negatively charged, promoting electrostatic repulsion and thereby reducing adsorption efficiency. Consequently, pH 3 was selected as the optimal pH for subsequent experiments, as it allows the coexistence of both ionic forms of the adsorbates, while maintaining a slightly positive surface charge on the AlAB_200W biochar, thereby favoring adsorption.

### 3.4 Effect of Temperature on Clz and Dic Adsorption on AlAB_200W

The effect of temperature on the adsorption of Clz and Dic was evaluated in the range of 298–328 K at an initial concentration of 60 mg L^–1^. As shown in Figure 8, the adsorption capacity of both pharmaceuticals increased progressively with increasing temperature, exhibiting an almost linear enhancement across the studied range. For instance, the adsorption capacity of Clz increased from 238.5 to 280 mg g^–1^, while the adsorption capacity of Dic increased from 250 to 285 mg g^–1^ as the temperature rose from 298 to 328 K.

**Figure 8:**
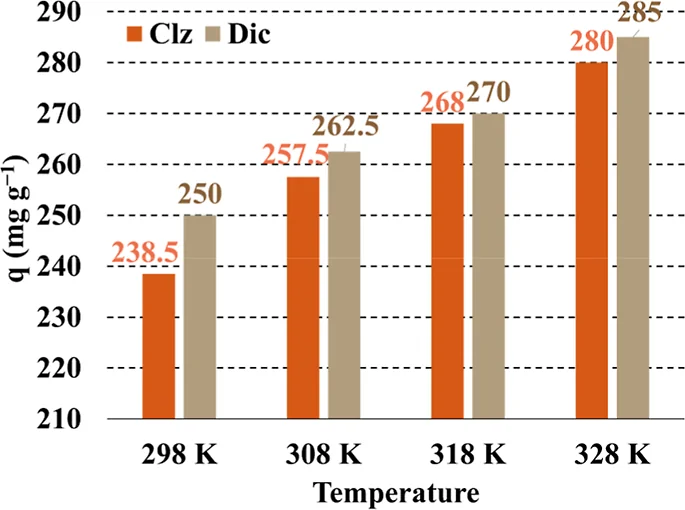
Effect of temperature on Clz and Dic adsorption.

This behavior highlights the strong thermally activated nature of the process^71^ and reflects the combined adsorption mechanisms governing the interaction between the pharmaceuticals and the AlAB_200W biochar, which involve both physisorption and surface complexation with the Al-coated functional groups (chemisorption-like contribution). Such mechanisms require a minimum activation energy to promote effective binding, which is facilitated at higher temperatures. As the temperature increases, the system provides sufficient thermal energy for more molecules to overcome interaction barriers and form stronger bonds or associations with the adsorbent surface. Additionally, an increased temperature enhances the mobility and diffusion coefficients of the pharmaceutical molecules, resulting in faster transport toward the active sites and a higher collision frequency with the adsorbent.^71^

### 3.5 Clz and Dic Adsorption Kinetics onto AlAB_200W

Evaluating the adsorption kinetics for the AlAB_200W biochar is essential to understand the removal mechanisms of Clz and Dic, as well as the influence of the 200 W modification on adsorption rate and control mechanisms.^72^ Figure 9 shows the experimental and predicted kinetic curves for the adsorption of Clz and Dic on AlAB_200W. The kinetic curves (*qt* versus time) exhibited the typical profile observed for liquid-phase adsorption systems, characterized by a rapid uptake stage followed by a slower phase until equilibrium was reached. This behavior indicates a high availability of active sites at the beginning of the process, followed by their gradual occupation until surface saturation of the AlAB_200W biochar.^51^ The adsorption equilibrium was achieved at approximately 120 min for Clz and 80 min for Dic.

**Figure 9:**
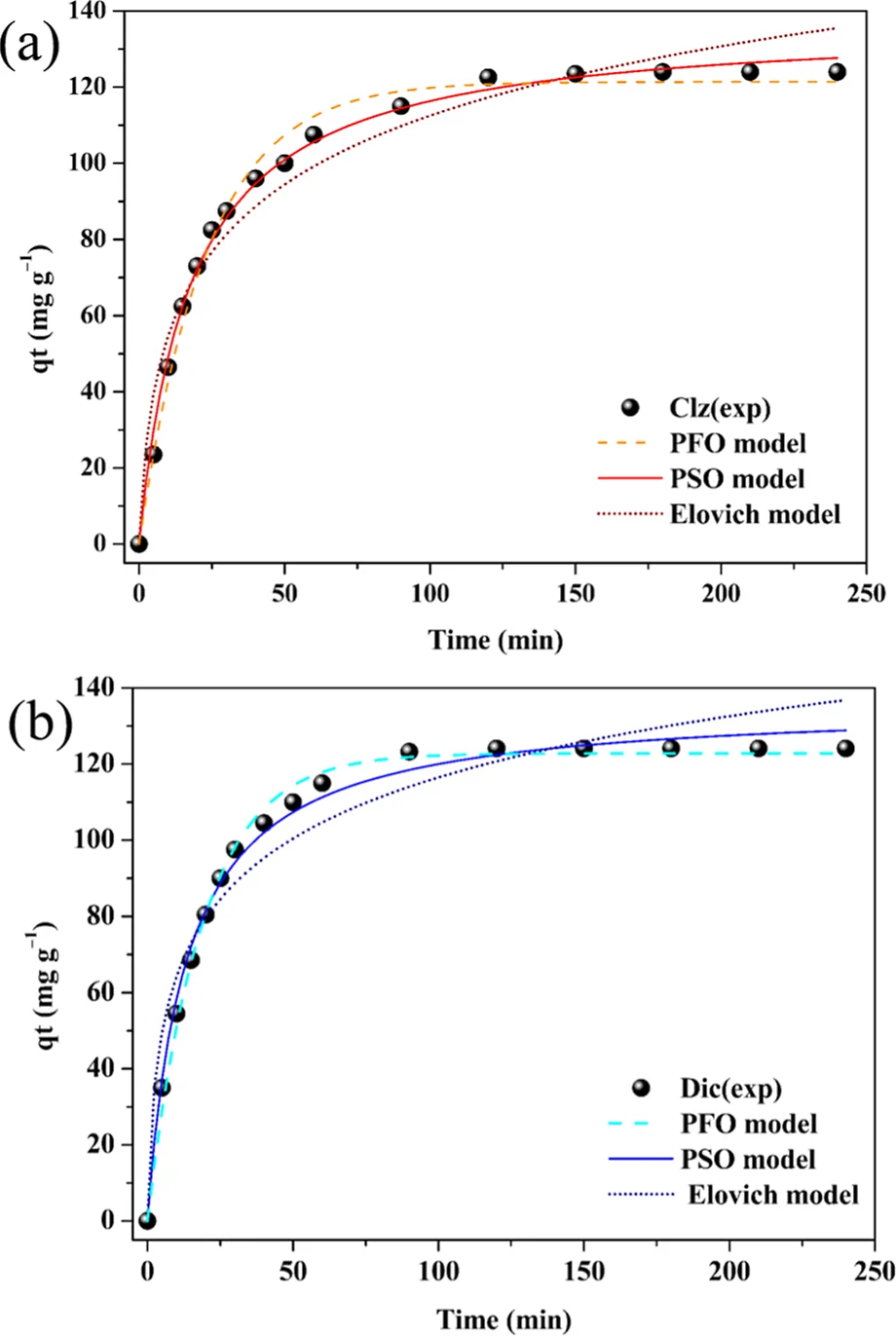
Adsorption kinetics curves of (a) Clz and (b) Dic.

The analysis of the kinetic model fittings (Table 2) shows that the PFO, PSO, and Elovich models all provided satisfactory fits, with *R* ^2^ and *R*_adj_^2^ values above 0.95 and low RMSE, confirming the good representativeness of the experimental data (Figure 9). Among them, the PSO model provided the best statistical fit (*R* ^2^ = 0.995 for Clz and 0.993 for Dic), indicating that it offers the most suitable empirical description of the adsorption kinetics under the investigated conditions. However, the PFO model also showed an excellent fit (*R* ^2^ > 0.99) and theoretical *q*_e_ values close to the experimental data, particularly for Dic, indicating that physisorption remains a relevant mechanism.^73,74^ The *k*_1_ value was higher for Dic (0.0536 min^–1^) than for Clz (0.0432 min^–1^), confirming faster adsorption of Dic. This behavior can be attributed to the smaller molecular size and orientation of Dic molecules, facilitating its movement until the active sites of AlAB_200W^75–77^ (Figure 9).

**Table 2:** Adsorption Kinetic Parameters of Clz and Dic on AlAB_200W

	pharmaceutical		
kinetic model	parameters	Clz	Dic
Pseudo-first-order	*k*_1_ (min^–1^)	0.0432	0.0536
	*q*_e_ (mg g^–1^)	121.4	122.8
	*R* ^2^	0.9915	0.9950
	*R*_adj_^2^	0.9906	0.9946
	RMSE	0.941	0.708
Pseudo-second-order	*k*_2_ (g mg^–1^ min^–1^)	0.000405	0.000551
	*q*_e_ (mg g^–1^)	137.2	136.0
	*R* ^2^	0.9957	0.9932
	*R*_adj_^2^	0.9954	0.9927
	RMSE	0.673	0.816
Elovich	α	18.05	33.9
	β	0.0376	0.0428
	*R* ^2^	0.9633	0.9474
	*R*_adj_^2^	0.9607	0.9437
	RMSE	7.36	2.28

The incorporation of the Elovich model provides further insight into the heterogeneity of the adsorption surface. The parameters α (18.05 mg g^–1^·min^–1^ for Clz and 33.9 mg g^–1^·min^–1^ for Dic) and β (0.0376–0.0428 g mg^–1^) reveal that the aluminum-modified biochar possesses a highly active and energetically diverse surface.^78^ The higher α value for Dic indicates a faster adsorption rate, consistent with the PSO kinetic constant. The moderate β values suggest a system where both physical and chemical adsorption mechanisms coexist.^78^

According to the highlighted feature section, the 200 W power modification significantly altered the biochar surface, changing the chemical surface and the density of oxygen-containing functional groups and introducing aluminum-related active sites capable of interacting with anionic pharmaceutical species. Therefore, the adsorption kinetics of AlAB_200W cannot be described by a single mechanism. While the PFO model adequately captures the initial fast physical adsorption stage, the Elovich model highlights energetic heterogeneity and chemical interactions at specific sites.^79^ In summary, the adsorption kinetics of AlAB_200W biochar are best interpreted as a hybrid process in which predominant physisorption is complemented by localized chemisorption, resulting in rapid attainment of equilibrium and high adsorption capacities for both pharmaceuticals.

### 3.6 Clz and Dic Adsorption Isotherms

The isotherms for Clz and Dic adsorption on AlAB_200W biochar are shown in Figure 10. The Langmuir, Freundlich, and Sips models were used to analyze the equilibrium data. The convex shape of the curves can be associated with L2-type, indicating extremely favorable adsorption, with a plateau indicating total occupation of the active sites of AlAB_200W biochar^80^ (Figure 10).

**Figure 10:**
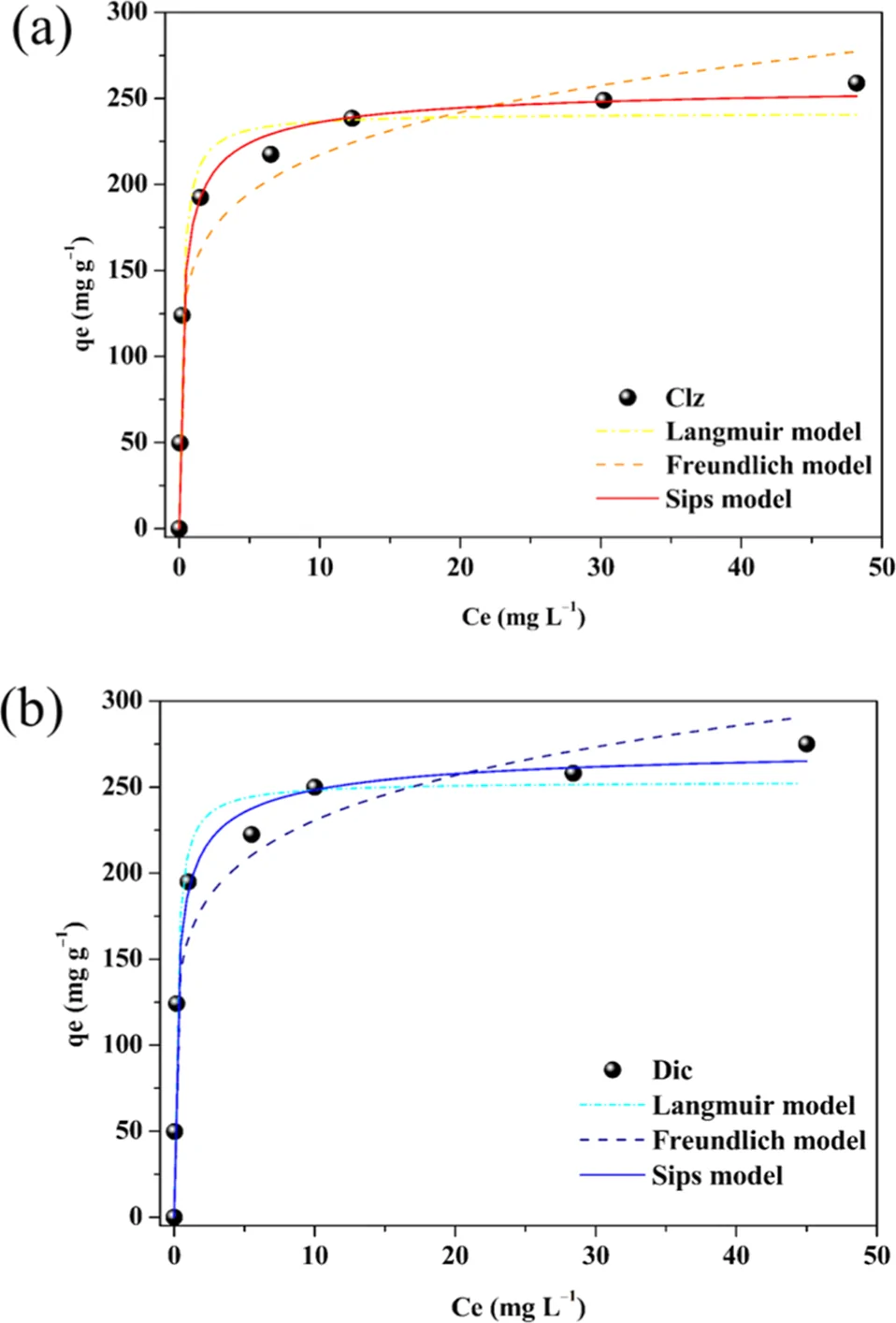
Adsorption isotherm curves of (a) Clz and (b) Dic on AlAB_200W.

Table 3 shows the isotherm parameters of Clz and Dic adsorption on AlAB_200W biochar. According to the statistical parameters, both Langmuir and Sips show a good fit (*R* ^2^, *R*_adj_^2^, and RMSE were 0.99, 0.99, and less than 10, respectively). The major differences were obtained when the temperature was increased. The Sips model presented slightly better values of *R* ^2^, *R*_adj_^2^, and RMSE (Table 3) than the Langmuir model at 298 K. Meanwhile, the Langmuir model showed higher values of *R* ^2^, *R*_adj_^2^, and RMSE at higher temperatures.

**Table 3:** Adsorption Isotherm Parameters of Clz and Dic on AlAB_200W at Four Temperatures

		temperature (K)							
		298		308		318		328	
isotherm		Clz	Dic	Clz	Dic	Clz	Dic	Clz	Dic
Langmuir	*K*_L_ (L mg^–1^)	4.67	5.03	4.76	5.28	6.52	7.03	7.6	10.75
	*q*_m_ (mg g^–1^)	241.6	277.2	266.2	278.3	274.1	289.4	298.9	306.2
	*R* ^2^	0.9848	0.9783	0.9588	0.9507	0.9473	0.9393	0.9208	0.9209
	*R*_adj_^2^	0.9804	0.9747	0.9515	0.9425	0.9386	0.9292	0.9076	0.9078
	RMSE	12.7	14.1	8.5	9.76	10.03	11.3	13.6	13.9
Freundlich	*K*_F_ (mg g^–1^/mg L^–1^)^−1/*n*^	152	161.9	166.9	176.9	178.4	191.3	200	212.2
	*n*	0.387	0.154	0.17	0.171	0.166	0.167	0.174	0.17
	*R* ^2^	0.8928	0.914	0.9626	0.9688	0.9622	0.9734	0.9742	0.9663
	*R*_adj_^2^	0.8391	0.871	0.9564	0.9636	0.9559	0.969	0.97	0.9607
	RMSE	7.49	23.9	8.1	7.76	8.5	7.51	7.74	9.12
Sips model	*K*_S_ (L mg^–1^)	**3.24**	**3.5**	**1.71**	**1.54**	**1.83**	**1.43**	**0.898**	**1.41**
	*q*_ms_ (mg g^–1^)	**260.6**	**277.5**	**327.3**	**356.9**	**348.3**	**393.5**	**437.8**	**442.8**
	*m*	**0.652**	**0.605**	**0.49**	**0.444**	**0.453**	**0.392**	**0.371**	**0.381**
	*R* ^2^	**0.9930**	**0.9904**	**0.9937**	**0.993**	**0.992**	**0.9925**	**0.9908**	**0.9853**
	*R*_adj_^2^	**0.9902**	**0.9880**	**0.9919**	**0.9905**	**0.9885**	**0.9896**	**0.9873**	**0.9794**
	RMSE	**3.40**	**7.90**	**3.64**	**4.06**	**4.33**	**4.35**	**5.05**	**6.60**

In addition, the adsorption capacity of the Sips model (*q*_ms_) increased at higher temperatures for both contaminants (Table 3). When the temperature increased from 298 to 328 K, the *q*_ms_ increased from 260 and 277 mg g^–1^ to about 437 and 443 mg g^–1^ for Clz and Dic, respectively. In contrast, although *q*_ms_ increased with increasing temperature, the constant *K*s decreased (3.24 to 0.898 L mg^–1^) for Clz and 3.5 to 1.41 L mg^–1^ for Dic. This apparent divergence suggests a slightly exothermic process that enhances molecular diffusion and improves the accessibility of internal active sites, thereby promoting higher uptake despite the reduced adsorbent–adsorbate affinity.^81^

In addition, the *m* parameter of the Sips model describes the heterogeneity of the process and can be related to the Freundlich *n* factor (Table 3). The values of the *m* and *n* parameters clearly show how the Al-coated biochar behaves as the temperature increases. At 298 K, both Clz and Dic display their highest *n* and *m* values (*n* = 0.387 and *m* = 0.652 for Clz; *n* = 0.154 and *m* = 0.605 for Dic), suggesting that adsorption initially takes place on the most uniform and energetically favorable sites created by the Al layer.^53,82^ As the temperature rises to 308 and 318 K, these parameters gradually decrease for both compounds (*n* = 0.170–0.166 and *m* = 0.490–0.453 for Clz; *n* = 0.171–0.167 and *m* = 0.444–0.392 for Dic) (Table 3). This drop indicates that the adsorbates begin to explore regions of the surface that are more irregular and exhibit wider energy distributions. At 328 K, *n* and *m* reached their lowest levels (*n* = 0.174 and *m* = 0.371 for Clz; *n* = 0.170 and *m* = 0.381 for Dic), showing that the higher temperature improves diffusion and allows access to increasingly heterogeneous sites.^52^

Table 4 summarizes a brief comparison of the AlAB_200W adsorbent with other modified materials reported in the literature in terms of adsorption capacity (*q*_m_) for the removal of Clz and Dic. Although the data were obtained under different experimental conditions, it is possible to observe that AlAB_200W stands out, consistently showing the highest adsorption capacities for Clz and Dic. For Clz, only one study was identified in the literature, highlighting the limited availability of adsorption data for this emerging pharmaceutical and emphasizing the relevance of the present work. AlAB_200W exhibited a markedly higher adsorption capacity for Clz (437.8 mg g^–1^) than Maghnite clay (50.0 mg g^–1^) under comparable initial concentrations. For Dic, although some adsorbents reported higher adsorption capacities, these studies were generally conducted under substantially different experimental conditions, including higher initial concentrations and adsorbent dosages, limiting direct comparison. Nevertheless, AlAB_200W achieved a competitive adsorption capacity (442.8 mg g^–1^) while requiring a relatively low adsorbent dosage (10 mg), demonstrating its effectiveness for pharmaceutical removal. These results confirm that AlAB_200W is a promising candidate in removing Clz and Dic from wastewater, highlighting the potential of the magnetron sputtering technique for producing high-performance aluminum-coated adsorbents.

**Table 4:** Adsorptive Performance Comparison between AlAB_200W and Other Modified Biochars and for the Removal of Clz and Dic

adsorbent	concentration (mg L^–1^)	adsorbent dosage (mg)	pH	temperature (K)	adsorption capacity (mg g^–1^)	reference
**Clz**						
Maghnite	25	10–80	4.6	295	50.0	^16^
**AlAB_200W**	25	10.0	3.0	328	437.8	this work
**Dic**						
MnFe_2_O_4_ functionalized biochar	250	6.25	4.0	298	352	^83^
GWBB_200W15 min	25	25	5.0	298	798.8	^84^
W-carboxylate-doped-activated carbon (OMWW)	400	-	4.0	298	711	^83^
BC1:2–900 biochar	100	10	4.0–10.0	288–318	684.2	^85^
magnetic gelatin-activated biochar	50	5.0	6.5	298	348.9	^86^
Fe-BC	10–150	10.0	5.0	288–318	361.2	^87^
**AlAB_200W**	25	10.0	3.0	328	442.8	this work

### 3.7 Clz and Dic Adsorption Thermodynamics

The adsorption curves of Clz and Dic on AlAB_200W under four temperatures (298–328 K) are shown in Figure 11. Both pharmaceuticals displayed a strong uptake at low equilibrium concentrations, followed by a progressive approach to saturation, indicating a strong affinity between the adsorbates and the active sites of the Al-modified biochar surface.

**Figure 11:**
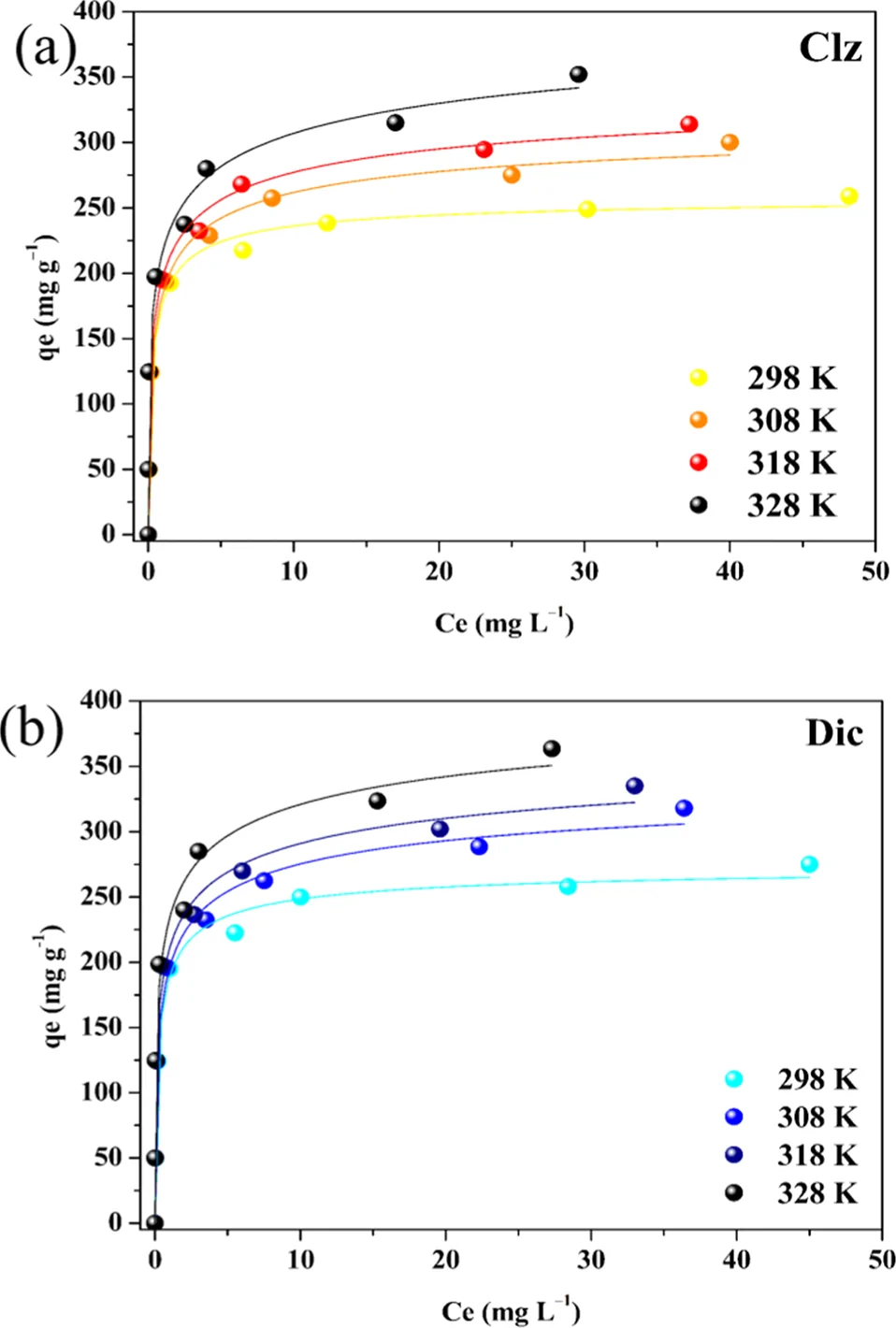
Adsorption curves for (a) Clz and (b) Dic under 298, 308, 318, and 328 K.

For both Clz and Dic, increasing the temperature from 298 to 328 K enhanced the adsorption capacity, with maximum *q*_e_ values exceeding 350 mg g^–1^ at 328 K. This behavior indicates that higher temperatures improve mass transfer and facilitate diffusion into the porous structure of AlAB_200W, allowing the pharmaceutical molecules to access adsorption sites that are less accessible at lower temperatures. Accordingly, the increase in adsorption capacity is mainly attributed to improved intraparticle diffusion rather than to an increase in the intrinsic affinity between the adsorbent and adsorbates.^88^

The negative Δ*G*° values (Table 5) confirm that the adsorption of both pharmaceuticals is spontaneous over the investigated temperature range.^71^ Although the equilibrium constant (ln *Ke*) decreases with increasing temperature, indicating a slight reduction in adsorbent–adsorbate affinity, adsorption remains thermodynamically favorable.^54^ This behavior is consistent with the Sips isotherm results, in which the affinity constant (*K*_s_) decreases while the maximum adsorption capacity (*q*_ms_) increases with temperature, demonstrating that improved accessibility of adsorption sites outweighs the reduction in equilibrium affinity.^54^

**Table 5:** Thermodynamic Parameters of Clz and Dic Adsorption

pharmaceuticals	AlAB_200W				
	*T* (K)	ln *K*_e_	Δ*G*° (kJ mol^–1^)	Δ*H*° (kJ mol^–1^)	Δ*S*° (J mol K^–1^)
Clz	298	6.92	–17.2	–32.1	50.8
	308	6.28	–16.1		
	318	6.18	–16.3		
	328	5.64	–15.4		
Dic	298	6.93	–17.2	–23.3	22.4
	308	6.12	–15.7		
	318	6.04	–15.9		
	328	6.02	–16.4		

The negative Δ*H*° values (−32.1 kJ mol^–1^ for Clz and −23.3 kJ mol^–1^ for Dic) indicate that the adsorption is exothermic, characteristic of adsorption dominated by noncovalent interactions.^71^ Therefore, the adsorption mechanism is mainly governed by electrostatic attraction, hydrogen bonding, π–π interactions, and metallic interactions involving surface Al–OH groups rather than by the formation of strong chemical bonds.

The positive Δ*S*° values indicate an increase in disorder at the solid–liquid interface during adsorption, which is commonly attributed to the release of structured water molecules from both the hydrated biochar surface and the hydration shells of the pharmaceutical molecules.^89^ This favorable entropy contribution partially compensates for the reduction in adsorption affinity at higher temperatures and, together with enhanced intraparticle diffusion, explains the higher adsorption capacities observed at elevated temperatures despite the moderately exothermic nature of the process.^71,90^

Comparatively, Dic exhibited slightly higher adsorption capacities than Clz, particularly at intermediate temperatures, which can be attributed to its greater hydrophobicity and molecular structure, favoring π–π interactions with the aromatic domains of the Al-coated biochar. Finally, the thermodynamic results indicate that adsorption on AlAB_200W is spontaneous and exothermic, while the improved adsorption performance at higher temperatures is primarily controlled by enhanced mass transfer and access to heterogeneous adsorption sites rather than by stronger adsorbent–adsorbate interactions.

### 3.8 Potential for the Treatment of Real Hospital Wastewater

The characteristics of the real hospital wastewater collected from a university hospital, and the initial concentration of the pharmaceutical compounds are shown in Table S1 of the Supporting Information. To further demonstrate the potential of AlAB_200W in the uptake of pharmaceutical compounds from real samples, adsorption assays were carried out using this hospital wastewater. The results of the removal percentages of each pharmaceutical compound from this effluent are shown in Table 6.

**Table 6:** Removal Percentage of Pharmaceutical Compounds from Real Hospital Wastewater

pharmaceutical compound	removal percentage (%)^a^	pharmaceutical compound	removal percentage (%)^a^
atenolol	–	ibuprofen	88.07 ± 1.89
atenolol acid	–	lidocaine	97.73 ± 0.69
benzotriazole	–	morphine	52.56 ± 1.63
carbamazepine	–	paracetamol	93.00 ± 1.27
ciprofloxacin	99.32 ± 0.52	propranolol	–
diazepam	–	sulfadiazine	–
diclofenac	94.75 ± 1.16	tramadol	–
fluconazole	–	venlafaxine	–

a Mean ± standard deviation (*n* = 3).

Hospital wastewater consists of a complex matrix that includes bacteria and pathogenic viruses, heavy metals, various classes of pharmaceutical compounds, and emerging micropollutants such as perfluorinated compounds, surfactants, personal care products, and hormones.^91^ Analgesic/anti-inflammatory drugs are among the most frequently detected classes of pharmaceuticals in these wastewaters.^92^ Indeed, as shown in Table S1, diclofenac (23.45 mg L^–1^), ibuprofen (12.32 mg L^–1^), and paracetamol (7.86 mg L^–1^) are the pharmaceuticals found at the highest concentrations in the collected wastewater. In addition, several other pharmaceutical compounds were detected at concentrations ranging from 0.04 mg L^–1^ to 2.95 mg L^–1^. The complex matrix and the large number of competitive compounds present in hospital wastewater can compete for active sites, reducing the affinity between the adsorbent surface and target compounds (diclofenac and other pharmaceutical compounds).

After treatment with the AlAB_200W adsorbent, TOC, NPOC, TN, TS, and COD decreased to 854 mg L^–1^, 1002 mg L^–1^, 152.3 mg L^–1^, 174.3 mg L^–1^, and 804 mg L^–1^, respectively, corresponding to reductions of 28.86%, 23.92%, 39.13%, 6.04%, and 14.98%. These results indicate that coexisting compounds may compete for the active sites of the adsorbent and be coadsorbed with the pharmaceutical compounds. In addition, such coexisting species can affect the density and viscosity of the solution, impacting the mass transfer of the pharmaceutical compounds, and can also affect the electrostatic interactions between the pharmaceutical molecules and the active sites of the adsorbent,^93^ hindering the adsorption of the target compounds. Despite this, the AlAB_200W adsorbent removed approximately 95% of diclofenac from the real hospital wastewater (Table 6). In addition, the AlAB_200W adsorbent efficiently removed other pharmaceuticals, including ciprofloxacin, ibuprofen, lidocaine, and paracetamol, with removal efficiencies exceeding 88%. After treatment with the AlAB_200W adsorbent, the total pharmaceutical concentration in the hospital wastewater decreased from approximately 50 mg L^–1^ to approximately 5 mg L^–1^. These results suggest the potential applicability of the Al-coated material for removing pharmaceutical compounds from complex matrix hospital wastewater.

### 3.9 Proposed Adsorption Mechanism

The adsorption performance of AlAB_200W for the removal of Clz and Dic results from the combined contribution of electrostatic attraction, hydrogen bonding, π–π interactions, and weak coordination interactions involving the aluminum coating. The relative importance of these mechanisms is corroborated by FTIR characterization, alongside pH-dependent adsorption behavior, equilibrium isotherms, and thermodynamic analysis.

FTIR spectra (Figure 2) revealed that the aluminum deposition modified the surface chemistry of the biochar without altering its carbonaceous structure. The broad band centered at approximately 3445 cm^–1^, attributed to O–H stretching vibrations, became more intense after modification, indicating a higher abundance of surface hydroxyl groups associated with oxidized aluminum species, a finding supported by the XPS analysis in Figure S2. Simultaneously, the band near 2900 cm^–1^, attributed to the CH_2_ stretching of graphitic domains of biochar, suggesting the participation of these groups in hydrogen bonding with oxygenated groups of adsorbates. The preservation of the aromatic vibration near 1580 cm^–1^ demonstrated that the graphitic domains remain intact, providing π-electron-rich regions capable of interacting with the aromatic rings of both drugs.^75^ These structural changes indicate that the aluminum coating primarily introduces additional polar adsorption sites while maintaining the aromatic structure responsible for noncovalent interactions.^60^

The pH-dependent adsorption behavior highlighted the importance of electrostatic interactions. Maximum adsorption occurred at pH 3, where the positively charged AlAB_200W surface (PZC = 6.0) favors interactions with partial deprotonated Clz and Dic species. These results indicate that electrostatic attraction primarily promotes the initial adsorption step, while hydrogen bonding and π–π interactions stabilize the adsorbed molecules (Figure 12).^33^

**Figure 12:**
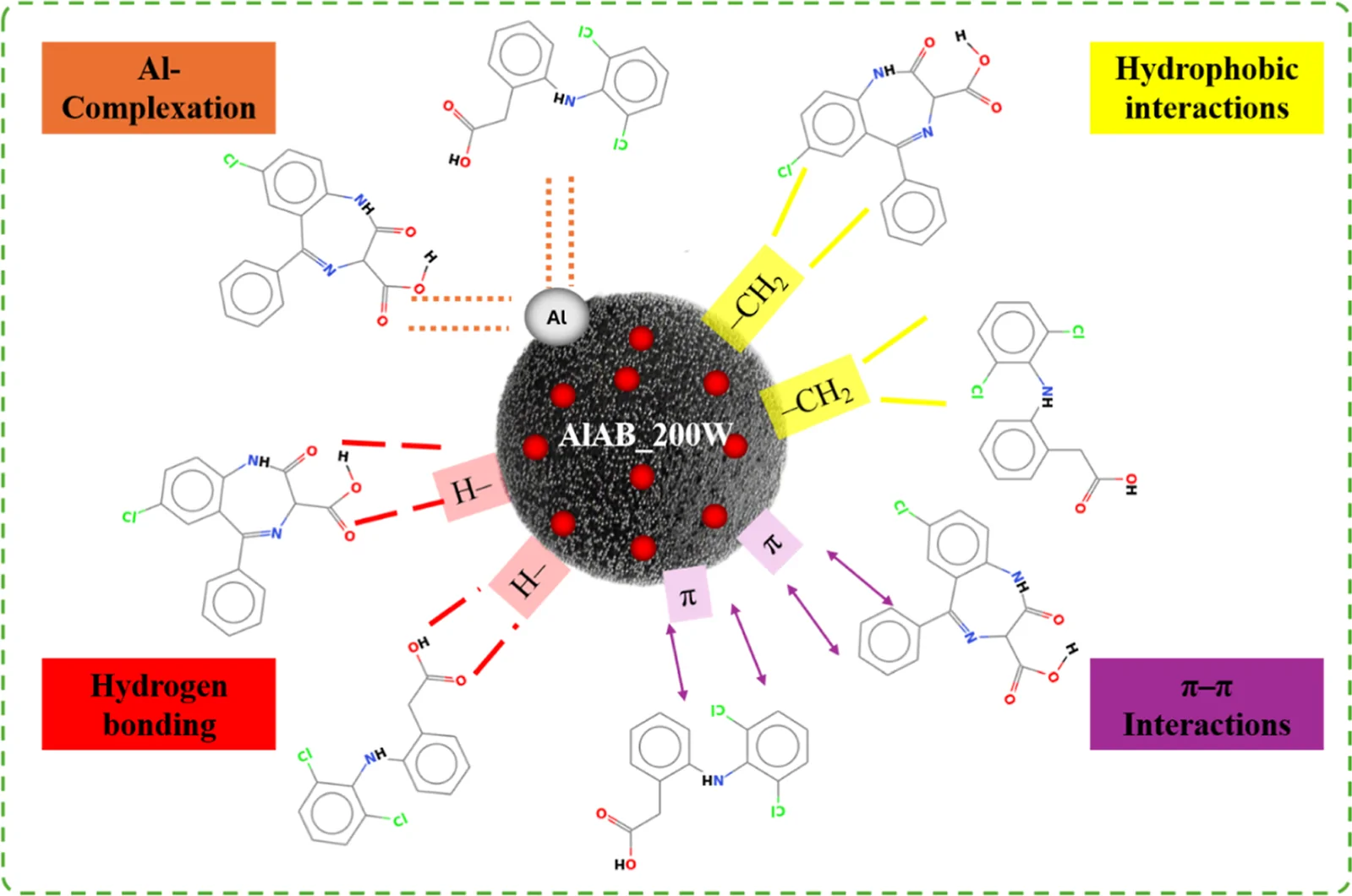
Proposed adsorption mechanism of Clz and Dic onto AlAB_200W biochar.

**13:**
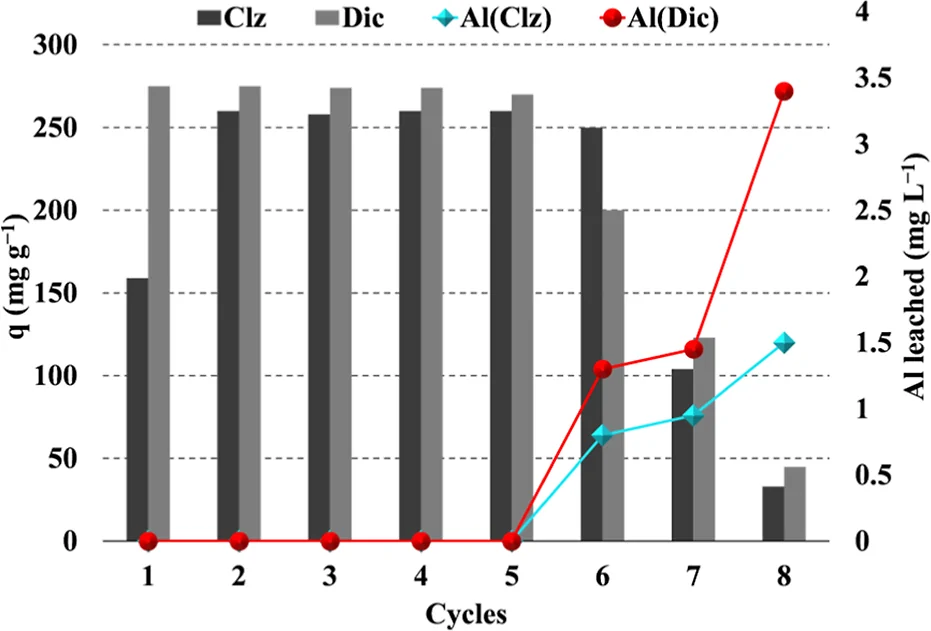
. Reusability of AlAB_200W biochar under 8 adsorption–desorption cycles.

Hydrogen bonding also contributes significantly to the adsorption process. The hydroxyl groups introduced by the aluminum coating can act as hydrogen bond donors or acceptors, interacting with the carboxyl, carbonyl, amino, and hydroxyl groups present in Clz and Dic^61^ (Figure 12). The increased intensity of the O–H stretching band following modification corroborates the higher density of hydrogen-bonding sites, which likely contributes to the superior adsorption capacity of AlAB_200W compared to untreated biochar.^46^ In addition to polar interactions, π–π electron donor–acceptor interactions contribute to the stabilization of the adsorbed molecules.^94^ These interactions are expected to be particularly relevant after the initial adsorption stage, increasing the stability of the adsorbed layer without requiring the formation of covalent bonds.^46,95^

The thermodynamic parameters further support the proposed mechanism. Negative Δ*G*° values confirm the spontaneous nature of adsorption, while negative Δ*H*° values indicate an exothermic process dominated by physical interactions, including electrostatic attraction, hydrogen bonding, and π–π interactions.^94^ The positive Δ*S*° values reflect increased disorder at the solid–liquid interface due to the release of hydration water, which, together with enhanced intraparticle diffusion, explains the higher adsorption capacities at elevated temperatures. Overall, Clz and Dic adsorption on AlAB_200W is governed by a cooperative mechanism in which electrostatic attraction promotes the initial approach of the molecules to positively charged Al sites, whereas hydrogen bonding and π–π interactions stabilize the adsorbed species.^84^ The heterogeneous distribution of these adsorption sites accounts for the high adsorption capacities and excellent performance of the Al-coated biochar (Figure 12).

The application of AlAB_200W to real hospital wastewater confirms the proposed adsorption mechanism. Although wastewater constituents compete for active sites and partially hinder interactions, the adsorbent remains highly effective, removing approximately 95% of Dic and over 88% of other pharmaceuticals, reducing total pharmaceutical concentration from 50 to 5 mg L^–1^. These results indicate that, although competitive adsorption occurs, the heterogeneous adsorption sites generated by the aluminum coating provide sufficient affinity toward aromatic pharmaceutical molecules through the combined action of electrostatic attraction, hydrogen bonding, and π–π interactions, maintaining high removal efficiency in a complex wastewater matrix.^6^

### 3.10 Regeneration and Reusability of AlAB_200W

The regeneration performance of the AlAB_200W biochar was evaluated to assess its stability and reusability (Figure 12). Under optimal adsorption conditions (Section 3.6), the material showed adsorption capacities of 260 mg g^–1^ and 277 mg g^–1^ for Clz and Dic at 298 K. During the first five regeneration cycles, AlAB_200W maintained stable performance with nearly constant adsorption values (250–270 mg g^–1^), indicating that the sputtered aluminum layer provided strong structural integrity and surface stability.

Aluminum leaching was monitored after each regeneration cycle by FAAS. No detectable aluminum was observed during the first five cycles, whereas concentrations of approximately 0.8, 1.0, and 3.4 mg L^–1^ were detected after the sixth, seventh, and eighth cycles, respectively. These results indicate that the Al coating remains stable during the initial reuse cycles but gradually dissolves under prolonged exposure to 0.5 mol L^–1^ NaOH. Consequently, the marked decline in adsorption capacity after the fifth cycle is attributed to the partial loss of the active aluminum layer. It is important to note that these concentrations correspond to the alkaline regeneration solution rather than to the treated effluent, and therefore, the spent regenerant should be properly managed before disposal. Therefore, AlAB_200W demonstrated excellent reliability and reusability under fifth regeneration cycles, ensuring efficient removal of drugs from aquatic waste.

## 4 Conclusion

In this study, a novel biochar derived from açaí waste and coated with aluminum via the magnetron sputtering technique (AlAB_200W) was applied for the removal of Clz and Dic from water and hospital wastewater. The AlAB_200W material exhibited pronounced changes in surface morphology and elemental composition after aluminum deposition. The effects of pH, temperature, and aluminum loading were systematically evaluated, revealing a strong dependence of adsorption performance on Al content and solution pH, while increasing temperature enhanced adsorption capacity. Kinetic data were best described by the pseudo-second-order model, indicating that the modified material involves both physical and chemisorption-related interactions, mainly associated with electrostatic attraction and surface complexation between aluminum sites and the anionic forms of the drugs. The Sips model best fitted the equilibrium data, and increasing the temperature from 298 to 328 K increased the maximum adsorption capacity (*q*_ms_) from 260 to 437.8 mg g^–1^ for Clz and from 278 to 442.8 mg g^–1^ for Dic. Thermodynamic analysis confirmed that the adsorption was spontaneous and favorable (negative Δ*G*°), and predominantly driven by physical interactions, as indicated by negative Δ*H*° and positive Δ*S*°. Experiments with real hospital wastewater demonstrated that AlAB_200W removed approximately 95% of Dic and more than 88% of other pharmaceuticals, reducing the total drug concentration from 50 to 5 mg L^–1^, thus confirming its high efficiency in complex matrices. Regeneration tests conducted over eight cycles showed a decline in performance from the sixth cycle onward, attributed to partial aluminum leaching. Compared with recent biochar-based adsorbents developed for the removal of Meropenem from aqueous systems, the present Al-coated biochar extends the applicability of engineered biochars by achieving the simultaneous removal of multiple pharmaceuticals from a complex hospital wastewater matrix, highlighting its potential for more realistic wastewater treatment scenarios.^21,96,97^ Finally, these findings highlight the strong potential of AlAB_200W as a versatile, cost-effective, and reusable adsorbent, representing a promising emerging material for water remediation applications.

## Supplementary Material



## Notes

SEM images of Raw biochar under 50 μm magnification; XPS analysis of AlAB_200W biochar; and hospital wastewater characteristics and concentration of pharmaceutical compounds in the hospital wastewater (PDF)

## References

[1] Leone V. O., Pereira M. C., Aquino S. F., Oliveira L. C. A., Correa S., Ramalho T. C., Gurgel L. V. A., Silva A. C. (2018). Adsorption of diclofenac
on a magnetic adsorbent based on maghemite: experimental and theoretical
studies. New J. Chem..

[2] Lai L. W., Teh L. P., Timmiati S. N., Kamarudin N. H. N., Setiabudi H. D. (2023). A sustainable solution for diclofenac adsorption: Chitosan-modified
fibrous silica KCC-1 adsorbent. J. Environ.
Chem. Eng..

[3] Verlicchi P., Al Aukidy M., Galletti A., Petrovic M., Barceló D. (2012). Hospital effluent:
Investigation of the concentrations and distribution of pharmaceuticals
and environmental risk assessment. Sci. Total
Environ..

[4] Majumder A., Gupta A. K., Ghosal P. S., Varma M. (2021). A review on hospital
wastewater treatment: A special emphasis on occurrence and removal
of pharmaceutically active compounds, resistant microorganisms, and
SARS-CoV-2. J. Environ. Chem. Eng..

[5] Parida V. K., Sikarwar D., Majumder A., Gupta A. K. (2022). An assessment
of
hospital wastewater and biomedical waste generation, existing legislations,
risk assessment, treatment processes, and scenario during COVID-19. J. Environ. Manage..

[6] Majumder A., Bhatnagar A., Kumar Gupta A. (2023). Simultaneous removal of sulfamethoxazole,
17β-estradiol, and carbamazepine from hospital wastewater using
a combination of a continuous constructed wetland-based system followed
by photocatalytic reactor. Chem. Eng. J..

[7] Teodosiu C., Gilca A.-F., Barjoveanu G., Fiore S. (2018). Emerging pollutants
removal through advanced drinking water treatment: A review on processes
and environmental performances assessment. J.
Clean. Prod..

[8] Montinari M. R., Minelli S., De Caterina R. (2019). The first
3500 years of aspirin history
from its roots—A concise summary. Vasc.
Pharmacol..

[9] Panchal N. K., Prince Sabina E. (2023). Non-steroidal
anti-inflammatory drugs (NSAIDs): A current
insight into its molecular mechanism eliciting organ toxicities. Food Chem. Toxicol..

[10] Jain B., Jain R., Nowak P. M., Ali N., Ansari M. N., Kabir A., Chandravanshi L. P., Sharma S. (2024). Comparison of various
sample preparation methods for benzodiazepines in terms of the principles
of white analytical chemistry. TrAC, Trends
Anal. Chem..

[11] Loftsson T. (2021). 1,4-Benzodiazepines:
Chemical stability and cyclodextrin solubilization. J. Drug Delivery Sci. Technol..

[12] Stefan M., Stefan I., Negoita I.-A., Ordeanu V., Stefan D. S. (2021). Influence
of Internal Structure of the Sorbents on Diazepam Sorption from Simulated
Intestinal Fluid. Appl. Sci..

[13] Barbieri I. A., Oliveira M. L. S., Bruckmann F. S., Salles T. R., Zancanaro L. V., Silva L. F. O., Dotto G. L., Lima E. C., Naushad Mu., Bohn Rhoden C. R. (2024). Effective removal of hypnotic drug from the aqueous
medium through adsorption on graphene oxide magnetic derivatives. J. Mol. Liq..

[14] Lai L. W., Teh L. P., Timmiati S. N., Kamarudin N. H. N., Setiabudi H. D. (2023). A sustainable solution for diclofenac
adsorption: Chitosan-modified
fibrous silica KCC-1 adsorbent. J. Environ.
Chem. Eng..

[15] Duarte E. D. V., Oliveira M. G., Spaolonzi M. P., Costa H. P. S., Silva T. L. d., Silva M. G. C. d., Vieira M. G. A. (2022). Adsorption of
pharmaceutical products from aqueous solutions on functionalized carbon
nanotubes by conventional and green methods: A critical review. J. Clean. Prod..

[16] Laouameur K., Flilissa A., Erto A., Balsamo M., Ernst B., Dotto G., Benguerba Y. (2021). Clorazepate
removal from aqueous
solution by adsorption onto maghnite: Experimental and theoretical
analysis. J. Mol. Liq..

[17] Kosjek T., Perko S., Zupanc M., Zanoški Hren M., Landeka Dragičević T., Žigon D., Kompare B., Heath E. (2012). Environmental occurrence, fate and
transformation of benzodiazepines in water treatment. Water Res..

[18] Salgado R., Marques R., Noronha J. P., Carvalho G., Oehmen A., Reis M. A. M. (2012). Assessing the
removal of pharmaceuticals and personal
care products in a full-scale activated sludge plant. Environ. Sci. Pollut. Res..

[19] Fent K., Weston A. A., Caminada D. (2006). Ecotoxicology of human
pharmaceuticals. Aquat. Toxicol..

[20] Sathishkumar P., Meena R. A. A., Palanisami T., Ashokkumar V., Palvannan T., Gu F. L. (2020). Occurrence, interactive
effects and
ecological risk of diclofenac in environmental compartments and biota
- a review. Sci. Total Environ..

[21] Duarte J. L. S., Hayat A., Domínguez C. M., Santos A., Cotillas S. (2025). Forest biomass
derived biochar for effective Meropenem mitigation in hospital effluents. J. Hazard. Mater. Adv..

[22] Kumari, A. ; Maurya, N. S. ; Tiwari, B. Hospital wastewater treatment scenario around the globe. In Current Developments in Biotechnology and Bioengineering; Elsevier, 2020; pp 549–570.

[23] Ouyang J., Zhou L., Liu Z., Heng J. Y. Y., Chen W. (2020). Biomass-derived
activated carbons for the removal of pharmaceutical mircopollutants
from wastewater: A review. Sep. Purif. Technol..

[24] Oulebsir A., Chaabane T., Tounsi H., Omine K., Sivasankar V., Flilissa A., Darchen A. (2020). Treatment of artificial
pharmaceutical
wastewater containing amoxicillin by a sequential electrocoagulation
with calcium salt followed by nanofiltration. J. Environ. Chem. Eng..

[25] Wakejo W. K., Meshesha B. T., Kang J. W., Dessalegn E. E., Demesa A. G. (2024). Integrated electrochemical-adsorption
for simultaneous
removal of pharmaceuticals from water: Process optimization and synergistic
insights. Chemosphere.

[26] Wang L., Bian Z. (2020). Photocatalytic degradation of paracetamol on Pd–BiVO4 under
visible light irradiation. Chemosphere.

[27] Bunmahotama W., Lin T.-F., Yang X. (2020). Prediction
of adsorption capacity
for pharmaceuticals, personal care products and endocrine disrupting
chemicals onto various adsorbent materials. Chemosphere.

[28] Wakejo W. K., Maged A., Meshesha B. T., Kang J. W., Demesa A. G., Chakrabarti S., Bhaskar T., Gupta A. K., Bhatnagar A. (2024). Tuneable functionalized
biochar for simultaneous removal of pharmaceuticals from binary mixture. Colloids Surf., A.

[29] Pauletto P. S., Lütke S. F., Dotto G. L., Salau N. P. G. (2021). Adsorption mechanisms
of single and simultaneous removal of pharmaceutical compounds onto
activated carbon: Isotherm and thermodynamic modeling. J. Mol. Liq..

[30] Sato M. K., de Lima H. V., Costa A. N., Rodrigues S., Pedroso A. J. S., de Freitas Maia C. M.
B. (2019). Biochar from Acai agroindustry
waste: Study of pyrolysis conditions. Waste
Manage..

[31] Sato M. K., de Lima H. V., Noronha
Costa A., Rodrigues S., Mooney S. J., Clarke M., Silva Pedroso A. J., de Freitas Maia C. M.
B. (2020). Biochar as a sustainable
alternative
to açaí waste disposal in Amazon, Brazil. Process Saf. Environ. Prot..

[32] Kayranli B., Bilen M., Seckin I. Y., Yilmaz T., Dinc A., Akkurt F., Simsek H. (2024). Peanut shell biochar
for Rhodamine
B removal: Efficiency, desorption, and reusability. Chemosphere.

[33] Chai W. S., Aslam A. A., Li X., Wu T., Pang C. H. (2025). Surface-engineered
biochar: Recent advances in modification strategies for environmental
remediation and energy applications. J. Environ.
Chem. Eng..

[34] Dong X., Chu Y., Tong Z., Sun M., Meng D., Yi X., Gao T., Wang M., Duan J. (2024). Mechanisms of adsorption and functionalization
of biochar for pesticides: A review. Ecotoxicol.
Environ. Saf..

[35] Cui T., Xie Y., Zhang M., Raise A. (2025). Tetracycline removal from aqueous
media and hospital wastewater using a magnetic composite of mango
lignocellulosic kernel biochar/MnFe2O4/Cu@Zn-BDC MOF. Int. J. Biol. Macromol..

[36] Xue H., Deng L., Kang D., Zhao Y., Zhang X., Liu Y., Chen H., Ngo H. H., Guo W. (2024). Advanced biochar-based
materials for specific antibiotics removal from hospital wastewater
via adsorption and oxidative degradation. J.
Environ. Chem. Eng..

[37] Ratnesh R. K., Singh M., Pathak S., Dakulagi V. (2020). Reactive magnetron
sputtered–assisted deposition of nanocomposite thin films with
tuneable magnetic, electrical and interfacial properties. J. Nanopart. Res..

[38] Atta S., NarendraKumar U., Kumar K. V. A. N. P.
S., Yadav D. P., Dash S. (2023). Recent Developments and Applications of TiN-Based Films Synthesized
by Magnetron Sputtering. J. Mater. Eng. Perform..

[39] Jung Y. S., Lee D. W., Jeon D. Y. (2004). Influence
of dc magnetron sputtering
parameters on surface morphology of indium tin oxide thin films. Appl. Surf. Sci..

[40] Viana G. C. C., Ratova M., Dimopoulou A. E., Redfern J., O’Dowd K., Kelly P. J., Pillai S. C., Amorim C. C. (2025). BiVO4 thin films
design via magnetron sputtering for water treatment: Antimicrobial
activity, photocatalytic efficiency, and toxicity assessment. Chem. Eng. Sci..

[41] Dutra P. R., Amorim C. C., Gastelois P. L., Grao M., Ratova M., Santos A. P., Kelly P. (2024). Carbonaceous-TiO2
composite photocatalysts
through reactive direct current magnetron sputtering on powdered graphene
for environmental applications. Thin Solid Films.

[42] Funari
Junior R. A., Oliveira Silva L. F., Silva Oliveira M. L., Godinho M., Aguzzoli C., Yousef Alomar S., Dotto G. L. (2025). Coating of Pinus elliottii-derived biochar with titanium
thin film via magnetron sputtering to enhance paracetamol adsorption. Chem. Eng. J..

[43] Kelly P. J., Arnell R. D. (2000). Magnetron sputtering:
a review of recent developments
and applications. Vacuum.

[44] Benvegnú I. A., Silva L. F. O., AlMasoud N., Alomar T. S., Crissien T., da Silva T. R., Aguzzoli C., Godinho M., Dotto G. L. (2025). Magnetron
sputtering-coated tungsten biochar: Efficient adsorption of Cu (II)
and methylene blue from wastewater. Mater. Chem.
Phys..

[45] Alberto A. L., Santos B. L. B. d., Godinho M., Aguzzoli C., Vieira Y., Silva L. F. O., Adelodun B., Mohandoss S., Ahmad N., Dotto G. L. (2025). Magnetron
sputtering of niobium thin
films onto wood biochar to increase the selectivity for electrostatic
attraction of anionic brilliant blue dye. J.
Taiwan Inst. Chem. Eng..

[46] Funari
Junior R. A., Fuhr A. C. F. P., Machado F. M., Oliveira M. L. S., Silva L. F. O., Knani S., Graba B., Dotto G. L. (2025). Mechanistic
and theoretical investigation of acetaminophen adsorption on titanium-coated
biochar: Statistical physics models and DFT insights. Surf. Interfaces.

[47] Pérez-González M., Morales-Luna M., Santoyo-Salazar J., Crotte-Ledesma H., García-Tinoco P. E., Tomás S. A. (2021). Improved
adsorption and photocatalytic removal of methylene blue by MoO3 thin
films: Role of the sputtering power, film thickness, and sputtering
working pressure. Catal. Today.

[48] Borowski P., Myśliwiec J. (2025). Recent Advances
in Magnetron Sputtering: From Fundamentals
to Industrial Applications. Coatings.

[49] Jadoon N. A. K., Puvanenthiram V., Mosa M. A. H., Sharma A., Wang K. (2024). Recent Advances in Aluminum Nitride (AlN) Growth by Magnetron Sputtering
Techniques and Its Applications. Inorganics.

[50] Hu Q., Pang S., Wang D. (2022). In-depth Insights into Mathematical
Characteristics, Selection Criteria and Common Mistakes of Adsorption
Kinetic Models: A Critical Review. Sep. Purif.
Rev..

[51] Qiu H., Lv L., Pan B., Zhang Q., Zhang W., Zhang Q. (2009). Critical review
in adsorption kinetic models. J. Zhejiang Univ.,
Sci., A.

[52] Ayawei N., Ebelegi A. N., Wankasi D. (2017). Modelling
and Interpretation of Adsorption
Isotherms. J. Chem..

[53] Piccin, J. S. ; Cadaval, T. R. S. ; de Pinto, L. A. A. ; Dotto, G. L. Adsorption Isotherms in Liquid Phase: Experimental, Modeling, and Interpretations. In Adsorption Processes for Water Treatment and Purification; Springer International Publishing: Cham, 2017; pp 19–51.

[54] Lima E. C., Hosseini-Bandegharaei A., Moreno-Piraján J. C., Anastopoulos I. (2019). A critical review of the estimation of the thermodynamic
parameters on adsorption equilibria. Wrong use of equilibrium constant
in the Van’t Hoof equation for calculation of thermodynamic
parameters of adsorption. J. Mol. Liq..

[55] Vieira Y., Pereira H. A., Leichtweis J., Mistura C. M., Foletto E. L., Oliveira L. F. S., Dotto G. L. (2021). Effective
treatment of hospital wastewater
with high-concentration diclofenac and ibuprofen using a promising
technology based on degradation reaction catalyzed by Fe0 under microwave
irradiation. Sci. Total Environ..

[56] Fan, M. ; Dai, D. ; Huang, B. Fourier Transform Infrared Spectroscopy for Natural Fibres. In Fourier Transform - Materials Analysis; InTech, 2012.

[57] Poliskie M., Clevenger J. O. (2008). Fourier
transform infrared (FTIR) spectroscopy for
coating characterization and failure analysis. Met. Finish..

[58] Sekine M., Sekiya H., Nakata M. (2012). Infrared and
Electronic Spectra of
Radicals Produced from 2-Naphthol and Carbazole by UV-Induced Hydrogen-Atom
Eliminations. J. Phys. Chem. A.

[59] Srinivasan P., Sarmah A. K., Smernik R., Das O., Farid M., Gao W. (2015). A feasibility study of agricultural and sewage biomass as biochar,
bioenergy and biocomposite feedstock: Production, characterization
and potential applications. Sci. Total Environ..

[60] You H., Lin H., Li Y., Yang Y., Ma Y., Shang Z., Niu X. (2023). Iron-aluminum
and aluminum-single impregnated biochar composite for
nitrate adsorption in rare earth wastewater: behavior and mechanism. Biomass Convers. Biorefin..

[61] Mwema F. M., Oladijo O. P., Akinlabi S. A., Akinlabi E. T. (2018). Properties
of physically
deposited thin aluminium film coatings: A review. J. Alloys Compd..

[62] Ahmed G., Mohamed W. S., Hasaneen M. F., Ali H. M., Ibrahim E. M. M. (2023). Optical,
structural, electrical and photocatalytic properties of aluminum doped
zinc oxide nanostructures. Opt. Mater..

[63] Sellaoui L., Dotto G. L., Pereira H. A., Vieira Y., dos Reis G. S., Oliveira M. L. S., Silva L. F. O., Khan M. R., Manoharadas S., Godinho M., Fantinel L. A., Aguzzoli C., Santos R. K. S. (2023). Adsorptive
properties of the pesticides 2,4-D, mecoprop, and dicamba on a pinus-based
biochar: Conventional and statistical physics evaluation. Chem. Eng. J..

[64] Leichtweis J., Silvestri S., Welter N., Vieira Y., Zaragoza-Sánchez P. I., Chávez-Mejía A. C., Carissimi E. (2021). Wastewater
containing emerging contaminants treated by residues from the brewing
industry based on biochar as a new CuFe2O4/biochar photocatalyst. Process Saf. Environ. Prot..

[65] Tillmann W., Lopes Dias N. F., Stangier D., Matveev S., Thomann C.-A., Debus J. (2021). Structure and tribo-mechanical properties
of Si- and W-containing
amorphous carbon based multilayers. Appl. Surf.
Sci. Adv..

[66] Rekkache H., Mezrag S., Fellah M., Bouras D., Bechiri L., Benslim N., Guesmi A., Khezami L. (2025). Characterization of
RF magnetron sputtered pure and aluminum-doped zinc oxide thin films
for optoelectronic applications. J. Mater. Sci.:Mater.
Electron..

[67] Saoula N., Djerourou S., Yahiaoui K., Henda K., Kesri R., Erasmus R. M., Comins J. D. (2010). Study of the deposition of Ti/TiN
multilayers by magnetron sputtering. Surf. Interface
Anal..

[68] Ladavos A. K., Katsoulidis A. P., Iosifidis A., Triantafyllidis K. S., Pinnavaia T. J., Pomonis P. J. (2012). The BET equation, the inflection
points of N2 adsorption isotherms and the estimation of specific surface
area of porous solids. Microporous Mesoporous
Mater..

[69] Muzyka R., Misztal E., Hrabak J., Banks S. W., Sajdak M. (2023). Various biomass
pyrolysis conditions influence the porosity and pore size distribution
of biochar. Energy.

[70] Vichaphund S., Wimuktiwan P., Soongprasit C., Soongprasit K., Phetchcrai S., Sirichaivetkul R., Atong D. (2023). Study on pyrolysis
behavior of polymeric coated aluminium scrap using TGA and Py-GC/MS. J. Anal. Appl. Pyrolysis.

[71] Salvestrini S., Ambrosone L., Kopinke F.-D. (2022). Some mistakes and misinterpretations
in the analysis of thermodynamic adsorption data. J. Mol. Liq..

[72] Adsorption Processes for Water Treatment and Purification; Bonilla-Petriciolet, A. , Mendoza-Castillo, D. I. , Reynel-Ávila, H. E. , Eds.; Springer International Publishing: Cham, 2017.

[73] Lee H., Fiore S., Berruti F. (2024). Adsorption of methyl orange and methylene
blue on activated biocarbon derived from birchwood pellets. Biomass Bioenergy.

[74] Ezzati R., Azizi M., Ezzati S. (2024). A theoretical
approach for evaluating
the contributions of pseudo-first-order and pseudo-second-order kinetics
models in the Langmuir rate equation. Vacuum.

[75] Wang Q., Zhang Z., Xu G., Li G. (2021). Pyrolysis
of penicillin
fermentation residue and sludge to produce biochar: Antibiotic resistance
genes destruction and biochar application in the adsorption of penicillin
in water. J. Hazard. Mater..

[76] Varela C. F., Moreno-Aldana L. C., Agámez-Pertuz Y. Y. (2024). Adsorption of pharmaceutical
pollutants on ZnCl2-activated biochar from corn cob: Efficiency, selectivity
and mechanism. J. Bioresour. Bioprod..

[77] Puga A., Moreira M. M., Pazos M., Figueiredo S. A., Sanromán M. Á., Delerue-Matos C., Rosales E. (2022). Continuous adsorption studies of pharmaceuticals in
multicomponent mixtures by agroforestry biochar. J. Environ. Chem. Eng..

[78] Wu F.-C., Tseng R.-L., Juang R.-S. (2009). Characteristics
of Elovich equation
used for the analysis of adsorption kinetics in dye-chitosan systems. Chem. Eng. J..

[79] Largitte L., Pasquier R. (2016). A review of the kinetics
adsorption models and their
application to the adsorption of lead by an activated carbon. Chem. Eng. Res. Des..

[80] Giles C. H., MacEwan T. H., Nakhwa S. N., Smith D. (1960). Studies in adsorption.
Part XI. A system of classification of solution adsorption isotherms,
and its use in diagnosis of adsorption mechanisms and in measurement
of specific surface areas of solids. J. Chem.
Soc..

[81] de
Vargas Brião G., Hashim M. A., Chu K. H. (2023). The Sips isotherm
equation: Often used and sometimes misused. Sep. Sci. Technol..

[82] Chen X., Hossain M. F., Duan C., Lu J., Tsang Y. F., Islam M. S., Zhou Y. (2022). Isotherm models for
adsorption of
heavy metals from water - A review. Chemosphere.

[83] Mechnou I., Oukkass S., El kartouti A., Hlaibi M., Saleh N., Alanazi A. K. (2025). High adsorption
capacity of diclofenac and paracetamol
using OMWW based W-carboxylate doped activated carbon. Sep. Purif. Technol..

[84] Lütke S. F., Funari Junior R. A., Biondo L. D., Aguzzoli C., Godinho M., Silva L. F. O., Oliveira M. L. S., Alves M. do C. M., Morais J., dos Reis G. S., Dotto G. L. (2026). Physical vapor deposition
of W on grape waste-based biochar and application of the material
as adsorbent for diclofenac, crystal violet, and Pb­(II): Effect of
deposition parameters on the material features and adsorption potential. Colloids Surf., A.

[85] Liu J., Zhang Y., Wang Y. (2026). Potassium
citrate-activated hierarchical
porous biochar from waste biomass: A promising adsorbent for diclofenac
removal with insights into adsorption mechanisms and column adsorption
behavior. Ind. Crops Prod..

[86] Thi
Minh Tam N., Liu Y.-G., Van Thom N. (2022). Magnetic gelatin-activated
biochar synthesis from agricultural biomass for the removal of sodium
diclofenac from aqueous solution: adsorption performance and external
influence. Int. J. Environ. Anal. Chem..

[87] Luo H., Zhang Y., Xie Y., Li Y., Qi M., Ma R., Yang S., Wang Y. (2019). Iron-rich
microorganism-enabled synthesis
of magnetic biocarbon for efficient adsorption of diclofenac from
aqueous solution. Bioresour. Technol..

[88] Souza P. R., Dotto G. L., Salau N. P. G. (2017). Detailed
numerical solution of pore
volume and surface diffusion model in adsorption systems. Chem. Eng. Res. Des..

[89] Funari R. A., Frescura L. M., de Menezes B. B., Bastos A. F. de M., da Rosa M. B. (2023). Adsorption of naphthalene and its
derivatives onto
high-density polyethylene microplastic: Computational, isotherm, thermodynamic,
and kinetic study. Environ. Pollut..

[90] Anastopoulos I., Kyzas G. Z. (2016). Are the thermodynamic parameters
correctly estimated
in liquid-phase adsorption phenomena?. J. Mol.
Liq..

[91] Ajala O. J., Tijani J. O., Salau R. B., Abdulkareem A. S., Aremu O. S. (2022). A review of emerging micro-pollutants
in hospital wastewater:
Environmental fate and remediation options. Results Eng..

[92] Santos L. H. M. L. M., Gros M., Rodriguez-Mozaz S., Delerue-Matos C., Pena A., Barceló D., Montenegro M. C. B. S. M. (2013). Contribution of hospital effluents
to the load of pharmaceuticals
in urban wastewaters: Identification of ecologically relevant pharmaceuticals. Sci. Total Environ..

[93] Nugraha M. W., Kim S., Roddick F., Xie Z., Fan L. (2025). A review of the recent
advancements in adsorption technology for removing antibiotics from
hospital wastewater. J. Water Process Eng..

[94] Tran H. N., Wang Y.-F., You S.-J., Chao H.-P. (2017). Insights
into the
mechanism of cationic dye adsorption on activated charcoal: The importance
of π–π interactions. Process
Saf. Environ. Prot..

[95] Fuhr A. C. F. P., Leichtweis J., Rossato D. L., Machado F. M., Knani S., Silva L. F. O., Crissien T. J., Dotto G. L. (2026). Statistical physics,
mechanisms, and reuse potential of an adsorbent from volcanic rock
powder towards metals and dyes. Mater. Res.
Bull..

[96] Duarte J. L. S., Hayat A., de la Fuente M., Domínguez C. M., Santos A., Cotillas S. (2025). Removal of Meropenem
from hospital
wastewater using biochar derived from grape stalks. J. Environ. Manage..

[97] Lima D. R., Hosseini-Bandegharaei A., Thue P. S., Lima E. C., de Albuquerque Y. R. T., dos Reis G. S., Umpierres C. S., Dias S. L. P., Tran H. N. (2019). Efficient
acetaminophen removal from
water and hospital effluents treatment by activated carbons derived
from Brazil nutshells. Colloids Surf., A.

