# Supplementary material for: Adsorption of Pharmaceutical Contaminants from a Real Hospital Effluent Using Açaí Biomass-Derived Biochar Modified via Magnetron Sputtering
Source: ACS Omega. 2026 Aug 18;11(34):51483–97. doi: 10.1021/acsomega.6c05238 (PMC13625516; doi:10.1021/acsomega.6c05238)
Supplement: Supplementary file 1 [file ao6c05238_si_001.pdf]

Adsorption of pharmaceutical contaminants from a real hospital effluent using açaí biomass-derived biochar modified via magnetron sputtering

Ronaldo Antunes Funari Junior[1], Sabrina Frantz Lütke[1], Luis Felipe Oliveira Silva[2*], Marcos Leandro Silva Oliveira[2], Alex Castro[3], Guilherme Luiz Dotto[1*]

[1]Universidade Federal de Santa Maria, Santa Maria, 97105-900, State of Rio Grande do Sul, BR.

[2]Corporación Universitaria de la Costa, Barranquilla, Colombia.

[3]Universidade Federal do Oeste do Pará, Santarem, 68035-110, Pará, BR.

[*]Corresponding authors: guilherme_dotto@yahoo.com.br; lfsoacademico@gmail.com

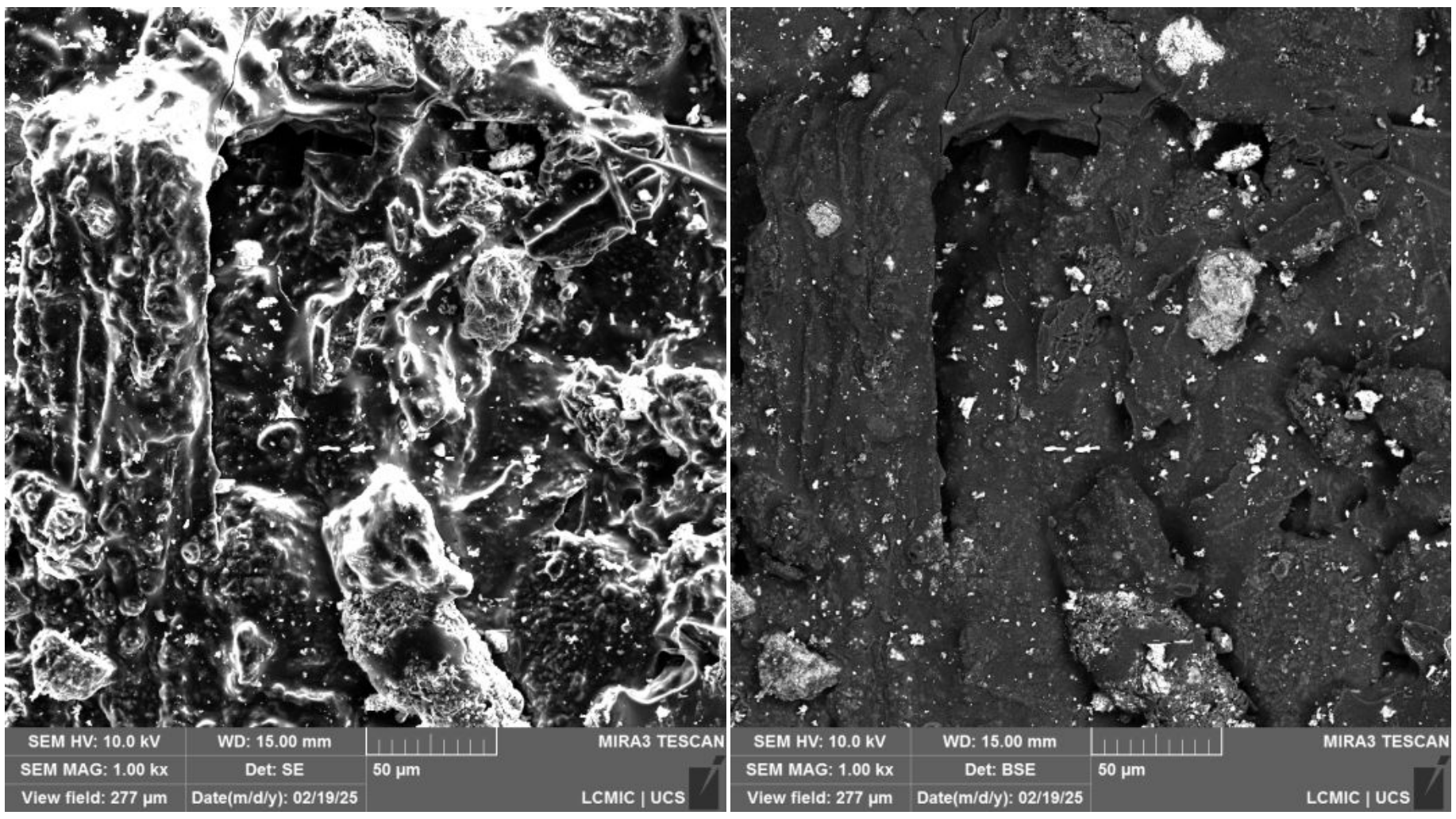

Fig.S1. SEM images of Raw biochar under 50 μm of magnification

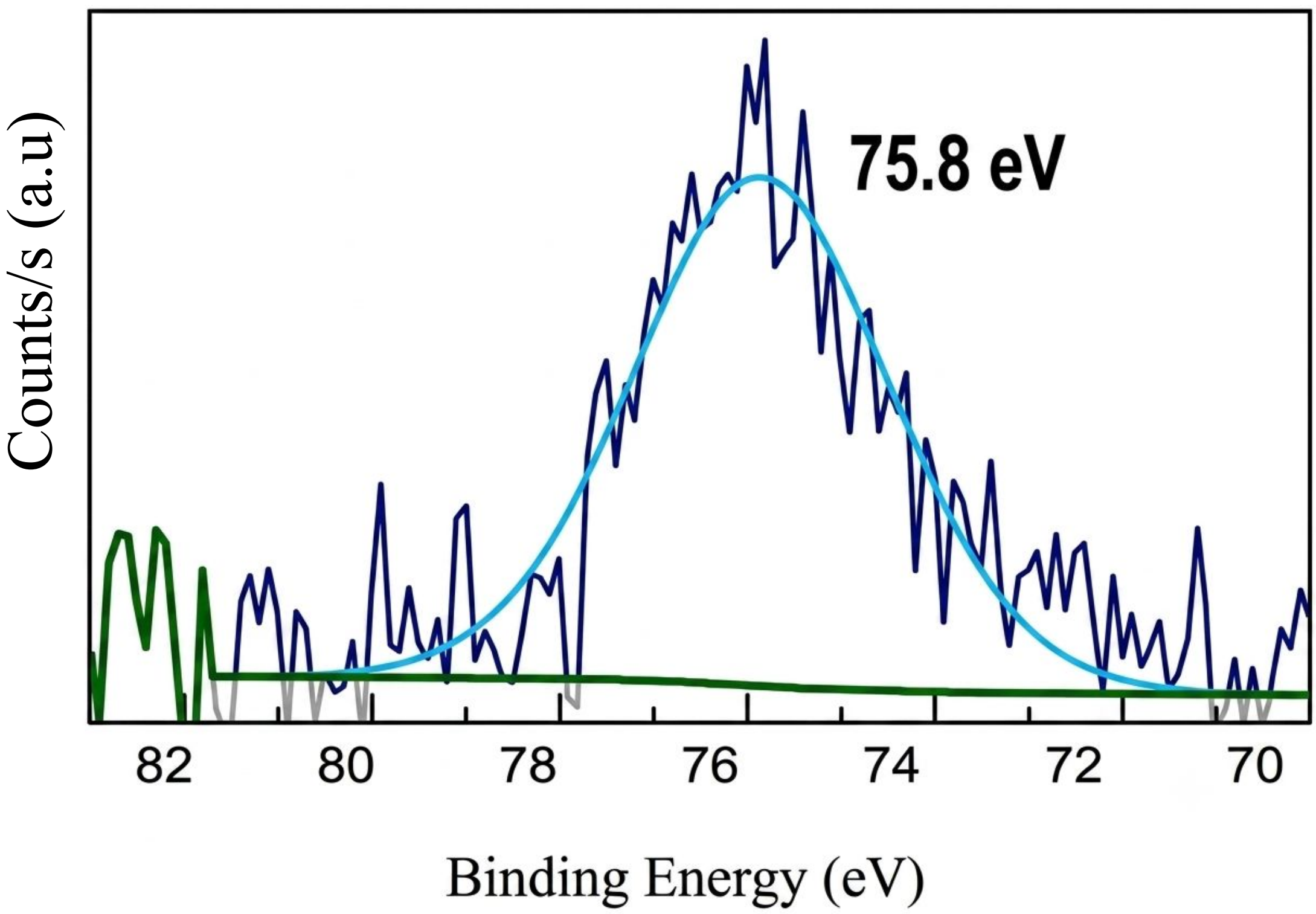

Fig. S2. XPS analysis of AlAB_200W biochar

Table S1. Hospital wastewater characteristics and concentration of pharmaceutical compounds in the hospital wastewater.

Parameter	Value
TOC	1200.5 ± 10.3 mg L^{-1}
NPOC	1317.7 ± 7.1 mg L^{-1}
TN	250.2 ± 2.5 mg L^{-1}
TS	185.5 ± 2.3 mg L^{-1}
COD	945.7 ± 8.3 mg L^{-1}
pH	5.1

**Pharmaceutical compound**	**Initial Concentration (mg L^{-1})***	**Pharmaceutical compound**	**Initial Concentration (mg L^{-1})***
Atenolol	0.185 ± 0.043	Ibuprofen	12.32 ± 0.012
Atenolol acid	0.799 ± 0.03	Lidocaine	2.21 ± 0.231
Benzotriazole	0.135 ± 0.06	Morphine	0.215 ± 0.122
Carbamazepine	0.04 ± 0.01	Paracetamol	7.86 ± 1.131
Ciprofloxacin	2.95 ± 0.02	Propanolol	0.05 ± 0.144
Diazepam	0.122 ± 0.042	Sulfadiazine	0.05 ± 1.263
Diclofenac	23.45 ± 0.055	Tramadol	0.085 ± 0.015
Fluconazole	0.125 ± 0.123	Venlafaxine	0.065 ± 0.003

Mean ± standard deviation ($n = 3$)
